# Hepatokine α1-Microglobulin Signaling Exacerbates Inflammation and Disturbs Fibrotic Repair in Mouse Myocardial Infarction

**DOI:** 10.1038/s41598-018-35194-w

**Published:** 2018-11-13

**Authors:** Daihiko Hakuno, Masahiro Kimura, Shinji Ito, Junko Satoh, Yasuhiro Nakashima, Takahiro Horie, Yasuhide Kuwabara, Masataka Nishiga, Yuya Ide, Osamu Baba, Hitoo Nishi, Tetsushi Nakao, Tomohiro Nishino, Fumiko Nakazeki, Satoshi Koyama, Ritsuko Hanada, Ruiz R. Randolph, Jin Endo, Takeshi Kimura, Koh Ono

**Affiliations:** 1 Department of Cardiovascular Medicine, Graduate School of Medicine, Kyoto University, 54 Kawaharacho, Shogoin, Sakyo-ku, Kyoto 606-8507 Japan; 2 Medical Research Support Center, Graduate School of Medicine, Kyoto University, 54 Kawaharacho, Shogoin, Sakyo-ku, Kyoto 606-8507 Japan; 3 Cardiovascular Division, Department of Internal Medicine, Keio University School of Medicine, 35 Shinanomachi, Shinjuku-ku, Tokyo 160-8582 Japan

## Abstract

Acute cardiac rupture and adverse left ventricular (LV) remodeling causing heart failure are serious complications of acute myocardial infarction (MI). While cardio-hepatic interactions have been recognized, their role in MI remains unknown. We treated cultured cardiomyocytes with conditioned media from various cell types and analyzed the media by mass spectrometry to identify α1-microglobulin (AM) as an Akt-activating hepatokine. In mouse MI model, AM protein transiently distributed in the infarct and border zones during the acute phase, reflecting infiltration of AM-bound macrophages. AM stimulation activated Akt, NFκB, and ERK signaling and enhanced inflammation as well as macrophage migration and polarization, while inhibited fibrogenesis-related mRNA expression in cultured macrophages and cardiac fibroblasts. Intramyocardial AM administration exacerbated macrophage infiltration, inflammation, and matrix metalloproteinase 9 mRNA expression in the infarct and border zones, whereas disturbed fibrotic repair, then provoked acute cardiac rupture in MI. Shotgun proteomics and lipid pull-down analysis found that AM partly binds to phosphatidic acid (PA) for its signaling and function. Furthermore, systemic delivery of a selective inhibitor of diacylglycerol kinase α-mediated PA synthesis notably reduced macrophage infiltration, inflammation, matrix metalloproteinase activity, and adverse LV remodeling in MI. Therefore, targeting AM signaling could be a novel pharmacological option to mitigate adverse LV remodeling in MI.

## Introduction

Acute myocardial infarction (MI) is a life-threatening coronary artery disease. While reperfusion therapy in the acute phase has significantly improved survival rate^1^, pharmacological intervention to mitigate cardiomyocyte necrosis has not been clinically evaluated, and serious complications, such as left ventricular (LV) free wall rupture, can occur in the acute phase of MI. Moreover, current pharmacological therapies do not entirely prevent adverse LV remodeling in the chronic phase, which increases the risk of heart failure (HF) after MI that is a major unresolved burden for MI patients worldwide^2^.

One of the regulators of MI pathology is inflammation, the extent and duration of which can alter a wide range of downstream processes. Indeed, multiple studies have shown that migrated neutrophils and classically activated macrophages (MQs) trigger inflammation and tissue destruction in addition to activating alternatively activated MQs and cardiac myofibroblasts for fibrotic repair and reducing inflammation^3–7^. MQs and cardiac fibroblasts (CFBs) interact with each other via cytokine, chemokine, and growth factor signaling during LV remodeling^7,8^ and affect the downstream Akt, NFκB, Stat3, and ERK1/2 signaling pathways that, in turn, regulate cell migration, inflammation, and fibrosis^7,9^. Interestingly, the upstream inflammatory signaling molecules have effects both locally and systemically, and inter-organ interactions during HF pathogenesis were recently observed^10^. For example, various secreted hepatokines, such as fetuin-A^11^, FGF-21^12^, and selenoprotein P^13^, affect glucose metabolism and chronic inflammation via the liver, adipocytes, and skeletal muscles. However, the direct effects of hepatokines on cardiac function during disease are largely unknown.

α1-microglobulin/bikunin precursor (AMBP) is a highly-conserved glycoprotein exclusively synthesized and secreted from the liver^14^. It is proteolytically processed into two different proteins: α1-microglobulin (AM) and bikunin (184 and 147 amino acids in human, respectively), which belong to the lipocalin family and the protease inhibitor family, respectively^15^. After secretion, AM is broadly distributed in the serum, monocytes, synovial fluid, cerebrospinal fluid, gut, kidneys, brain, heart, skin, liver, etc. and is excreted from the kidneys^14^. Functionally, AM is a heme-binding antioxidant protein that has been shown to inhibit heme-induced intracellular oxidation in cultured cells^16,17^ and reduces structural damage in hemoglobin- or heme-induced rat kidney injury and ewe preeclampsia models^18,19^. However, a recent study has questioned the utility of AM as a universal antioxidant as administration not only fails to decrease non-heme-induced injury, but worsens renal injury both *in vitro* and *in vivo*^20^. This controversy highlights our limited understanding of AM hepatokine function, receptor interaction, and the downstream signaling pathways affected during disease, particularly during cardiovascular inflammation.

In this study, we screened the effects of conditioned media from various cell types on stressed cardiomyocytes *in vitro* and identified AM as the primary component responsible for the effects observed following treatment with hepatocyte-derived conditioned media. Using a mouse MI model, we also demonstrate that AM treatment enhances MQ infiltration and CFB/MQ-mediated inflammation while inhibiting fibrotic repair, resulting in acute cardiac rupture. Furthermore, a protein-lipid overlay assay and lipid pull-down assay indicate that AM interacts with phosphatidic acid (PA), a functionally diverse phospholipid found in the plasma membrane that is involved in the progression of multiple disorders. Disruption of this interaction with PA synthesis inhibitors suggests that AM signaling is mediated by PA, and the administration of a selective inhibitor of PA synthesis had cardioprotective effects in mouse MI.

## Results

### α1-microglobulin activates Akt in cardiomyocytes

**Figure 1:**
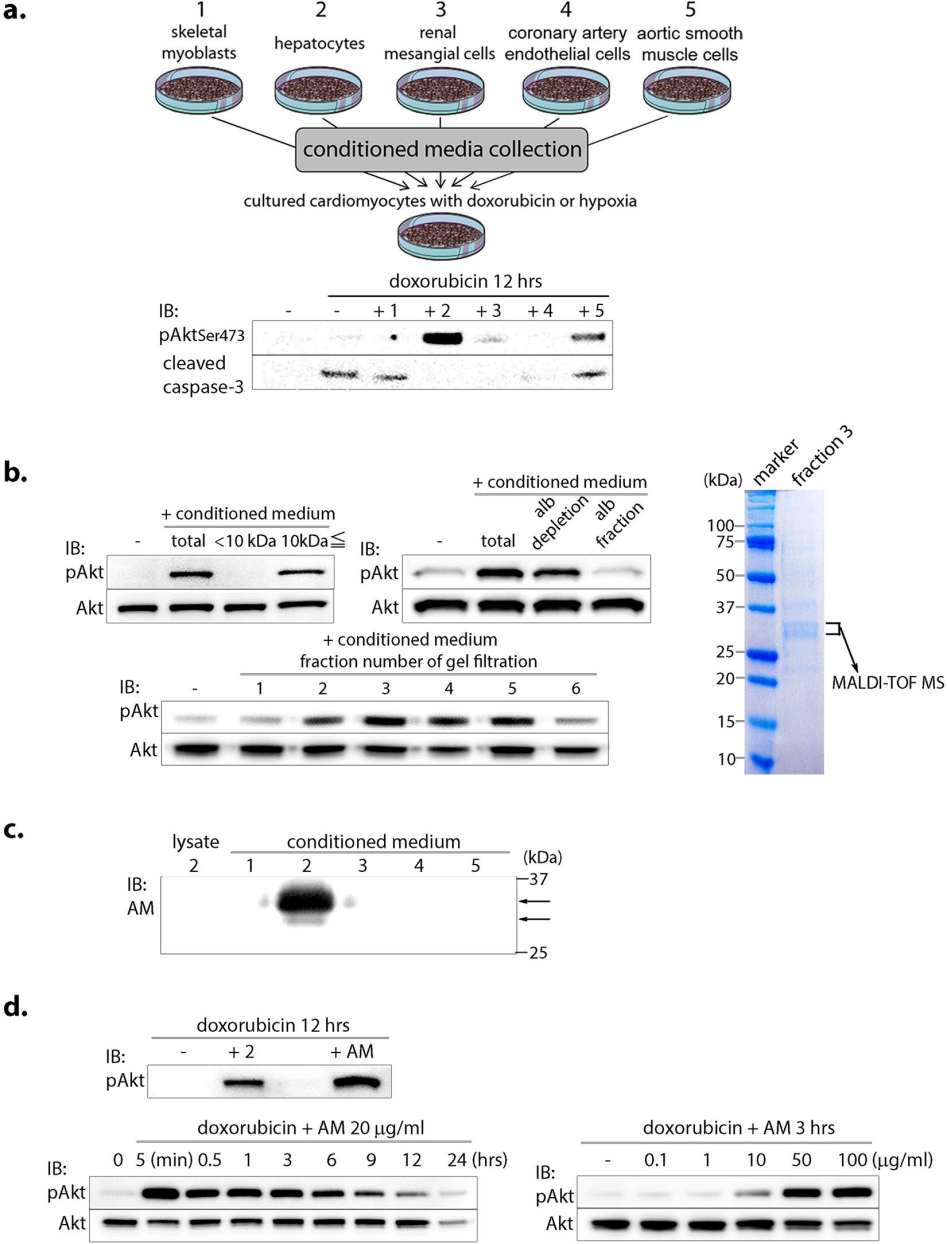
*In vitro* screening identified α1-microglobulin (AM) as an Akt-activating hepatokine in stressed cardiomyocytes. (**a**) (upper) Schematic diagram highlighting the cell types (labeled 1–5) used to evaluate the effects of conditioned media on primary cardiomyocytes under doxorubicin- or hypoxia-induced stress. (lower) Western blot analysis of Akt activation and caspase-3 inhibition in cardiomyocytes treated with doxorubicin and the specified conditioned medium (1–5). (**b**) Identification of AM/bikunin precursor in hepatocyte-derived conditioned medium by ion exchange chromatography, albumin depletion, gel filtration, and mass spectrometry. (**c**) Expression of AM/bikunin precursor protein in the cell lysate and the above conditioned media (1–5). The two bands (upper and lower) show the glycosylated and unglycosylated protein, respectively. (**d**) Akt activation in cardiomyocytes with AM treatment. Alb, albumin; dox, doxorubicin.

To investigate the inter-organ interactions functioning during cardiovascular disease, an *in vitro* screening was performed with conditioned media isolated from various cell types, including skeletal myoblasts, hepatocytes, renal mesangial cells, coronary artery endothelial cells, and aortic smooth muscle cells. This medium was then used to stimulate cultured cardiomyocytes during stress (doxorubicin- or hypoxia-induced) and the effects on function, namely Akt signaling and apoptosis, were evaluated. Of all the types investigated, stimulation with Huh7 hepatocyte-derived conditioned medium most strongly caused sustained Akt activation while inhibiting caspase-3 expression and apoptosis in cardiomyocytes under stress with doxorubicin (Fig. 1a) or hypoxia (1% O_2_, Supplementary Fig. S1a). A similar change in Akt activation was also observed in cardiomyocytes treated with primary hepatocyte-derived conditioned medium (Supplementary Fig. S1b).

Upon analyzing the components of the conditioned media, Akt activation appears to be mediated by a more than 10-kDa heat-sensitive protein that is not bound to albumin (Fig. 1b and Supplementary Fig. S1c). Following ion exchange chromatography, albumin depletion, and gel filtration (Fig. 1b and Supplementary Fig. S1d), mass spectrometry was finally used to identify the Akt-activating factor in Huh7-derived conditioned media as AMBP (Supplementary Table S1). Western blot analysis of the conditioned media confirmed that AMBP protein is secreted exclusively from hepatocytes (Fig. 1c). Although *AMBP* mRNA sequentially encodes both AM and bikunin, only stimulation with AM, not bikunin, activated Akt in cardiomyocytes (Supplementary Fig. S1e). Further, this effect was dose-dependent (Fig. 1d).

In wild type mice, *AM* mRNA is expressed in the liver, while protein is merely detected in the LV (Supplementary Fig. S1f). Notably, this expression was not affected by gender.

### AM is transiently distributed in the infarct and border zones during the acute phase of MI in mice

**Figure 2:**
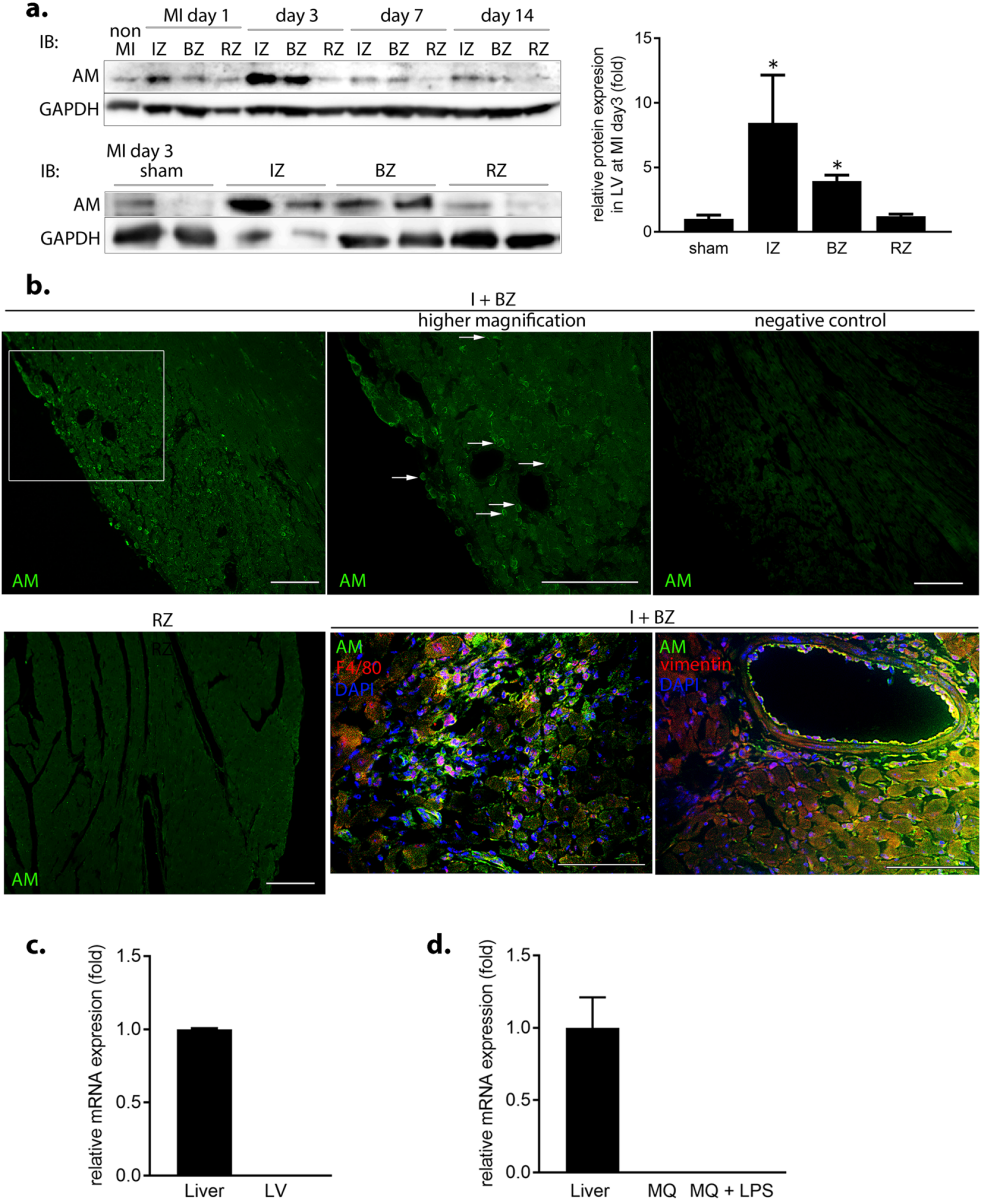
AM is transiently distributed in the infarct and border zones via infiltrated macrophages (MQs) and cardiac fibroblasts (CFBs) during the acute phase of mouse MI. (**a**) Serial and spatial AM protein expression in the LV. *p < 0.05, compared to sham (n = 4 per group). IZ, infarct zone; BZ, border zone; RZ, remote (non-MI) zone. (**b**) (upper) AM protein expression (green, arrows) in the infiltrated cells and interstitium of I + BZs at day 3 during MI. Negative control is a staining without the primary antibody. Scale bars, 100 μm. (lower) Representative immunofluorescent staining with AM (green) and F4/80 or vimentin (red) in the BZ. Scale bars, 60 μm. (**c**,**d**) Relative *AM* mRNA expression in the LV (**c**) and cultured MQs (**d**) compared to that in the liver.

Next, we investigated serial and spatial expression and distribution of AM protein in a mouse MI model. AM protein was slightly detected in the LV of sham-operated mice, but was transiently expressed in the infarct and border zones (I + BZs) of MI mice (Fig. 2a), with levels increasing from early on day 1, peaking at 8-fold higher on day 3, and returning to baseline by day 7. Moreover, AM protein was expressed in the infiltrated cells and interstitium both at the epicardial side (arrows in Fig. 2b) and endocardial side of the I + BZs at day 3. AM staining also appears to be co-localized with the MQ marker F4/80 and vimentin, but not with the granulocyte marker LY-6G^21^, indicating AM protein expression in CFBs and infiltrated MQs (Fig. 2b and Supplementary Fig. S2a). However, *AM* mRNA expression was undetected in the LV of wild type mice (Fig. 2c), and its expression was unchanged in the I + BZs of MI mice compared to that in the LV of sham mice during the acute phase. mRNA and protein expression in the liver and serum concentration of AM did not significantly differ between sham and MI mice. Furthermore, little to no mRNA was detected in cultured MQs with or without lipopolysaccharide stimulation (Fig. 2d). In accordance with the result in our mouse MI model, AM serum concentrations were also unchanged between control subjects without cardiovascular diseases and acute MI patients (Supplementary Table S2 and Fig. S2b).

These findings strongly suggest that AM binds to MQs after secretion from the liver and that the transient increase in AM protein distribution reflects infiltration of MQs and interstitial AM accumulation in the I + BZs during the acute phase of MI.

### AM enhances MQ migration as well as the proinflammatory response in CFBs and MQs *in vitro*

**Figure 3:**
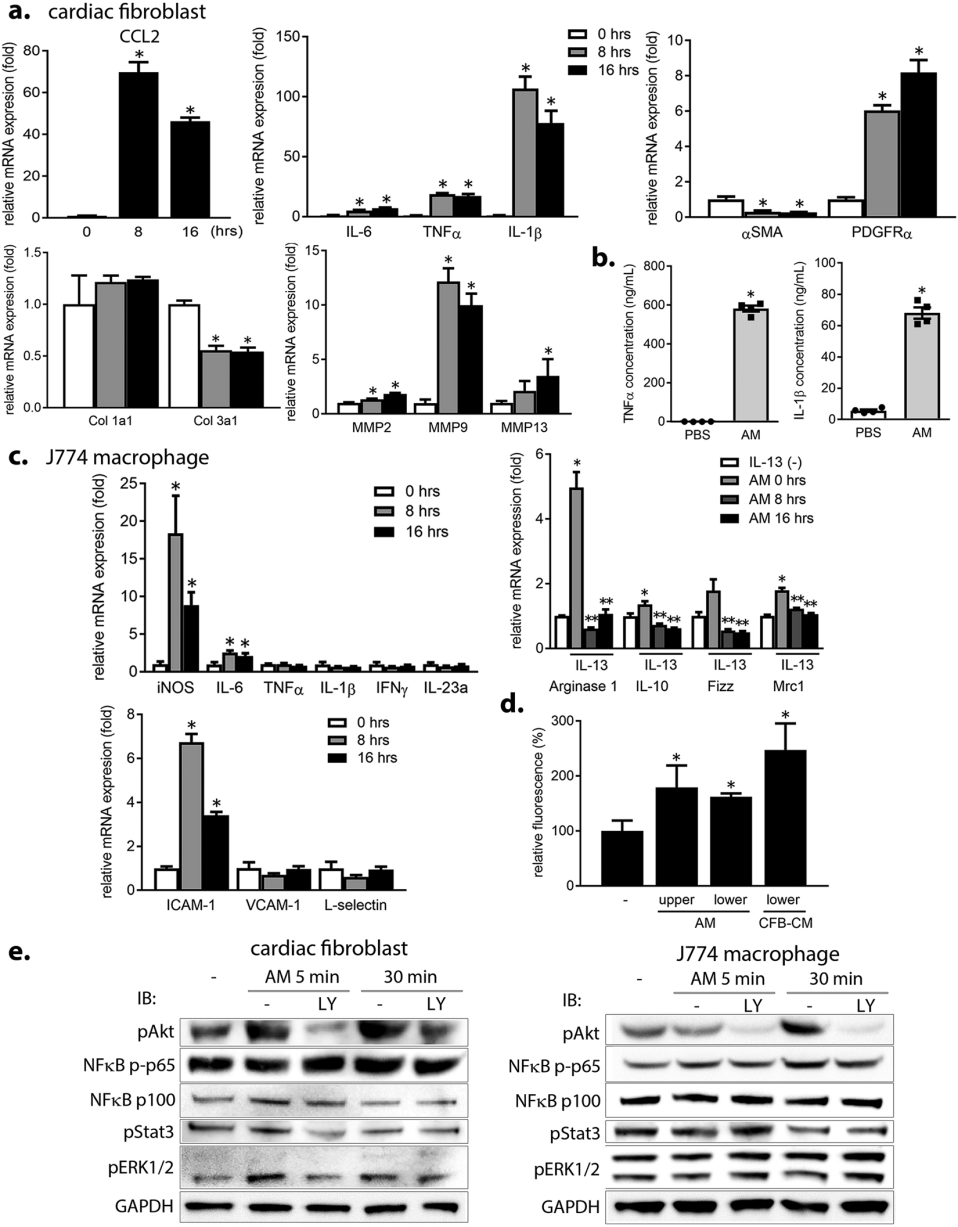
AM administration enhances MQ migration and inflammation in CFBs and MQs *in vitro*. (**a**) Changes in the mRNA expression in CFBs after AM administration. *p < 0.05, compared to 0 hrs. Col, collagen. (**b**) Changes in cytokine secretion from CFBs after AM stimulation. Concentration of TNFα and IL-1β in CFB-derived conditioned medium was analyzed by ELISA. *p < 0.05, compared to PBS. (**c**) Changes in the mRNA expression of proinflammatory cytokines, markers for the alternatively activated phenotype (arginase 1, IL-10, Fizz, Mrc1) under IL-13 stimulation, and cell adhesion molecules in MQs after AM administration. Mrc1, mannose receptor C-type 1. *p < 0.05, compared to 0 hrs or IL-13 (−). **p < 0.05, compared to AM 0 hrs with IL-13 stimulation. (**d**) MQ migration assayed using a modified Boyden chamber, whereby upper and lower labels indicate the upper and lower chambers, respectively. Serum-starved MQs were plated on the upper chambers, and human AM protein (20 μg/ml) was added to serum-free medium either on the upper or lower chambers. CFB-derived conditioned medium was used as a positive control. *p < 0.05, compared to the negative control. CFB-CM, CFB-derived conditioned medium. (**e**) AM-mediated activation of signaling pathways in CFBs and MQs. LY, LY294002 (phosphoinositide 3-kinase inhibitor).

To further understand the effects of AM on the function of other cell types during MI, including CFBs, MQs, and endothelial cells, these cells were cultured and treated with AM *in vitro*. In primary CFBs, AM treatment increased the mRNA expression of the major MQ chemotactic factor C-C motif chemokine ligand 2 (CCL2; 70-fold) and the proinflammatory cytokines IL-6, TNFα, and IL-1β (7~107-fold) (Fig. 3a) followed by secretion of these cytokines from the cells (Fig. 3b). AM administration also decreased the mRNA expression of fibrosis-related genes, such as α smooth muscle actin (αSMA) and collagen 3a1 (27% and 54%, respectively), while increasing that of platelet-derived growth factor receptor α and matrix metalloproteinases (MMPs) by 8-fold and 2~12-fold, respectively (Fig. 3a and Supplementary Fig. S3a). Notably, AM did not affect CFB proliferation.

In cultured MQs (J774.1 cell line), AM stimulation significantly augmented the mRNA expression of proinflammatory cytokines, such as inducible nitric oxide synthase (iNOS) and IL-6, whereas almost completely inhibited IL-13-induced increase in markers for alternatively activated MQs, such as arginase 1, IL-10, Fizz, and Mrc1 (Fig. 3c). Furthermore, AM administration increased intercellular adhesion molecule (ICAM)-1 and α4 integrin (Fig. 3c and Supplementary Fig. S3b). AM stimulation also significantly promoted MQ migration (Fig. 3d) and cell aggregation (Supplementary Fig. S3c) *in vitro*. The effect of AM on the mRNA expression of iNOS, IL-6, and ICAM-1 was more evident in bone marrow-derived macrophages than in J774.1 MQs (Supplementary Fig. S3d).

In human umbilical vein endothelial cells, AM administration increased *ICAM-1* and *E-selectin* mRNA expression (Supplementary Fig. S3e). However, AM did not affect angiogenesis (Supplementary Fig. S3f).

We next analyzed how AM administration mediates these effects by investigating Akt, NFκB, Stat3, and ERK1/2 signaling pathway activation in CFBs and MQs. In CFBs, Akt and ERK were activated as early as 5 min after AM stimulation, which was inhibited by phosphoinositide 3-kinase inhibitor LY294002, whereas NFκB signaling was not activated (Fig. 3e, left). On the other hand, in MQs, Akt and NFκB p65 were modestly activated at 30 min after AM stimulation, which was inhibited by LY294002 (Fig. 3e, right). AM stimulation did not activate Stat3 signaling in CFBs and MQs. These findings indicate that the signaling pathways activated with AM stimulation are different between in CFBs and in MQs.

### Intramyocardial AM administration augments MQ infiltration and inflammation, impairs fibrosis, and triggers acute cardiac rupture in mouse MI

**Figure 4:**
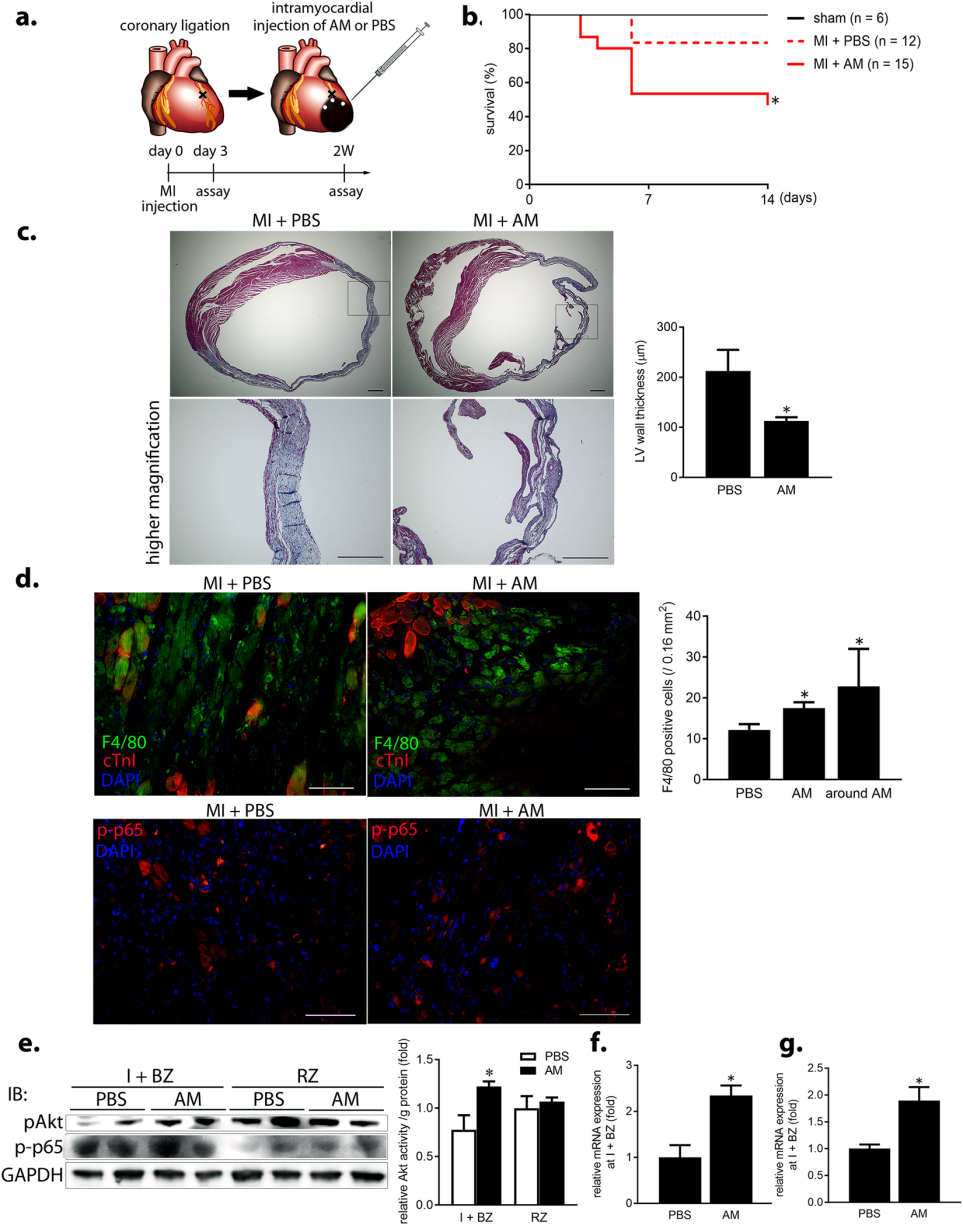
Intramyocardial AM administration augments MQ infiltration and inflammation, impairs fibrosis, and triggers acute cardiac rupture in mouse MI. (**a**) Schematic diagram showing intramyocardial administration in mouse MI, whereby PBS or diafiltrated recombinant mouse AM protein is injected into the BZ. (**b**) Survival curve of each treatment group. *p < 0.05, compared to the MI + PBS group (Log-rank test). (**c**) Masson’s trichrome staining at 2 weeks post-treatment. Scale bars, 500 μm and 300 μm (higher magnification). (**d**) (upper) Infiltration of F4/80 (green)-positive MQs and (lower) NFκB p65 activation (red) in the BZ at day 3. Scale bars, 60 μm. cTnI, cardiac troponin I. (**e**) (left) Akt activation in the I + BZs of the MI + AM group at day 3. (right) Densitometric analysis of Akt activity. *p < 0.05, compared to the I + BZs of the MI + PBS group (n = 4). (**f**,**g**) *MMP9* (f) and *iNOS* (**g**) mRNA expression in the I + BZs at day 3. *p < 0.05, compared to the MI + PBS group.

To verify the deleterious effect of AM on repair processes during MI, changes in LV phenotype were analyzed following intramyocardial AM protein administration in a mouse MI model. A preliminary experiment shows that human AM protein remained in the BZ for at least 2 days after injection (Supplementary Fig. S4a). Then, either recombinant murine AM protein or PBS (control) was injected at three sites in the BZ just after coronary artery ligation using a previously described method^22^ (Fig. 4a). Preservatives and endotoxins in the recombinant murine AM protein were removed by diafiltration prior to injection. Surprisingly, AM injection caused premature death due to acute cardiac rupture more frequently than PBS, with a 47% mortality rate compared to 17% at day 6, respectively (Fig. 4b and Supplementary Fig. S4b).

Using echocardiography, we found that E/e’, an index of LV end-diastolic pressure and pulmonary congestion in HF, was significantly increased at 2 weeks post-injection in the MI + AM group compared to that in the control group, without apparent changes in LV diameter or systolic function (Supplementary Fig. S4c). Histological analysis revealed that collagen fibril formation was impaired and the LV wall was significantly thinner at 2 weeks post-injection in the MI + AM group (Fig. 4c).

Next, the acute effects of AM during MI were examined at 3 days post-injection. The degree of F4/80-positive MQ infiltration and NFκB p65 activation in the BZ was higher in the MI + AM group compared to that in the control group (Fig. 4d). Furthermore, AM administration augmented Akt activation in the I + BZs (Fig. 4e). In support of these data, *MMP9* and *iNOS* mRNA expression was significantly increased in the I + BZs of the MI + AM group (Fig. 4f,g).

### AM strongly binds to membrane PA and partly signals via PA

To explore the signaling mechanism underlying AM function, a shotgun proteomic analysis was conducted to identify possible AM-receptor interactions in primary CFBs. A total of 13 proteins were enriched in the plasma membrane following AM stimulation (Supplementary Table S3). Of these, annexin A2 and vimentin were considered the most likely candidates to be AM receptors. However, while immunocoprecipitation confirmed AM binding to annexin A2, the binding with vimentin was not clearly detected (Supplementary Fig. S5a). Furthermore, when annexin A2 protein was blocked or its expression was modulated by siRNA or overexpression in CFBs, AM function was not altered, as highlighted by consistent IL-1β mRNA expression (Supplementary Fig. S5b,c).

**Figure 5:**
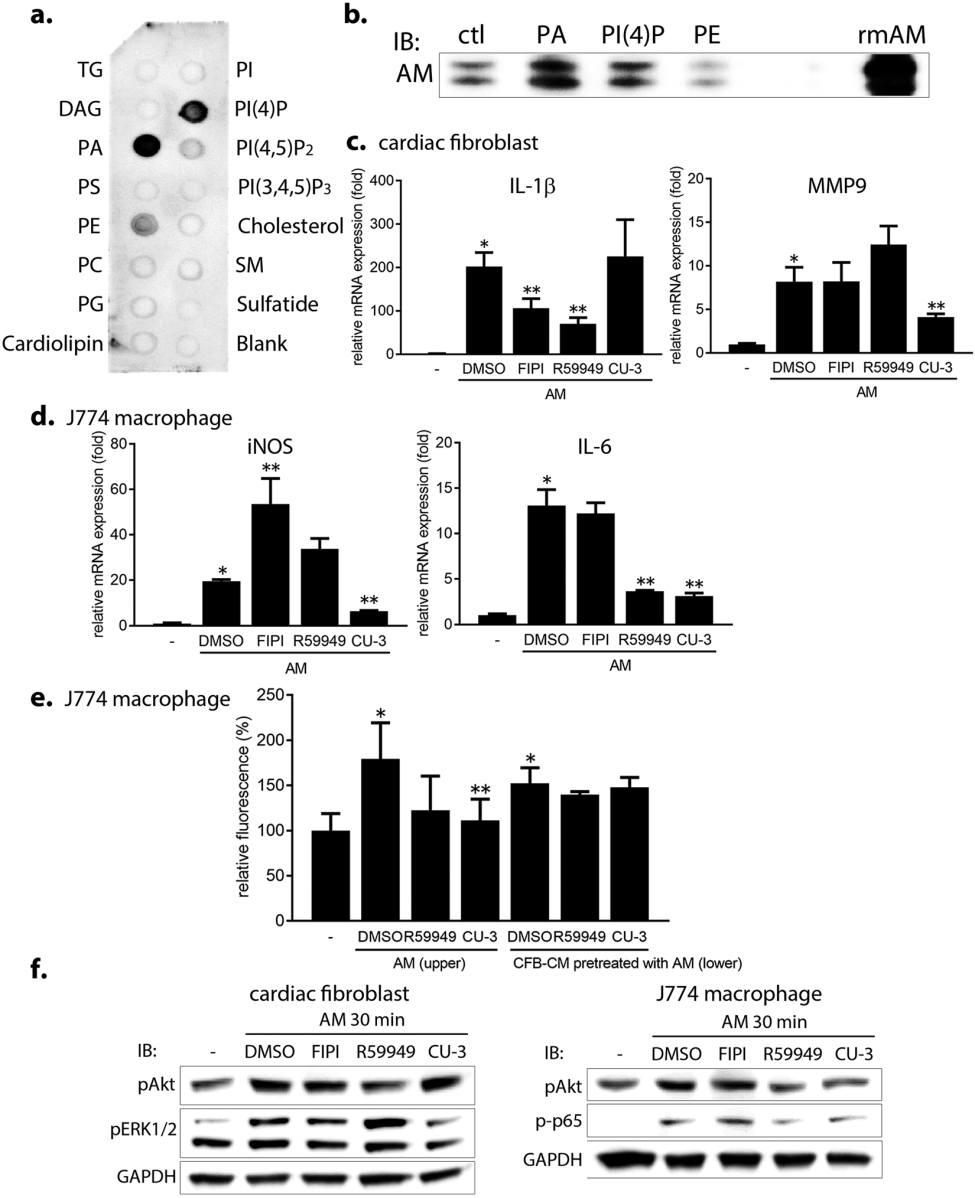
AM strongly binds to membrane PA and partly signals via PA. (**a**) Protein-lipid overlay assay using a membrane lipid strip. The lower right spot is a negative control (Blank). TG, triglyceride; DAG, diacylglycerol; PA, phosphatidic acid; PS, phosphatidylserine; PE, phosphatidylethanolamine; PC, phosphatidylcholine; PG, phosphatidylglycerol; PI, phosphatidylinositol; PI(4)P, phosphatidylinositol (4)-phosphate; PI(4,5)P_2_, phosphatidylinositol (4,5)-diphosphate; PI(3,4,5)P_3_, phosphatidylinositol (3,4,5)-triphosphate; SM, sphingomyelin. (**b**) Lipid-protein pull-down assay using lipid coated beads. The beads coated either with PA, PI(4)P, or PE were incubated with recombinant murine AM protein, pelleted by centrifugation, and subjected to a western blot using anti-mouse AM antibody. ctl, beads without lipid; rmAM, recombinant murine AM protein without beads (as a positive control). (**c**) Changes in mRNA expression in AM-stimulated CFBs with PA synthesis inhibitors. *p < 0.05, compared to the negative control; **p < 0.05, compared to AM + DMSO. (**d**,**e**) Changes in mRNA expression (**d**) and migration (**e**) of AM-stimulated MQs. CFB-CM, CFB-derived conditioned medium. (**f**) Changes in Akt, ERK1/2, and NFκB activation in AM-stimulated CFBs and MQs.

AM, also known as protein heterogeneous in charge, is a lipocalin family of protein, and has a biophysical or biochemical retentive mechanism by the lipocalin pocket^14^ that can trap small hydrophobic compounds. This information led us to hypothesize that it could bind to a plasma membrane phospholipid during signal transduction. To test this, a protein-lipid overlay assay was first performed with a membrane lipid strip and recombinant mouse AM protein to screen AM-lipid interactions. Surprisingly, our results showed that AM strongly binds to several glycerophospholipids in the plasma membrane such as PA, phosphatidylinositol (4)-phosphate (PI(4)P), and phosphatidylethanolamine (Fig. 5a). In contrast, AM did not bind to lysophosphatidic acid or to sphingophospholipids, another type of membrane phospholipid family that includes sphingosine-1-phosphate, ceramide, and sphingomyelin (Supplementary Fig. S5d). To further validate the interaction of AM and the screened lipids, a lipid-protein pull-down assay was performed with lipid coated beads and confirmed AM binding to PA (Fig. 5b).

PA is a major second messenger of signaling and synthesized by three enzymes: phospholipase D, diacylglycerol kinase (DGK), and 1-acylglycerol-3-phosphate O-acyltransferase (AGPAT)^23,24^. Therefore, to examine whether PA actually mediates AM signaling, we interfered with AM-PA interaction by inhibiting PA biosynthesis via these three enzymes and monitored the effects. In CFBs, AM-induced increase in *IL-1β* mRNA expression was significantly inhibited by pretreatment with either the phospholipase D1/D2 inhibitor FIPI^25^ or the non-selective DGK inhibitor R59949^26^, whereas increase in *MMP9* mRNA expression was inhibited by the selective DGKα inhibitor CU-3^27^ (Fig. 5c). These findings imply that phospholipase D and each DGK isoforms may activate different signaling pathways in CFBs. Furthermore, pretreatment none with AGPAT1 or AGPAT2 siRNA (Supplementary Fig. S5e,f) or simultaneous AGPAT1 and AGPAT2 siRNAs (Supplementary Fig. S5g) did not mitigate AM-induced changes in mRNA expression.

In MQs, pretreatment with CU-3 showed a significant decrease in iNOS and IL-6 expression (Fig. 5d). Moreover, AM-triggered MQ migration was decreased following direct pretreatment either with the DGK inhibitor R59949 or CU-3 (Fig. 5e).

Concordant to changes in mRNA expression and MQ migration with PA synthesis inhibitors, AM-activated Akt or ERK signaling was inhibited by pretreatment with R59949 or CU-3, respectively in CFBs (Fig. 5f). In contrast, AM-activated Akt and NFκB p65 signaling were partly inhibited both with R59949 or CU-3. These data suggest that AM signaling is partly mediated by PA.

### Short-term, systemic delivery of a selective DGKα inhibitor mitigates acute inflammation and adverse LV remodeling in mouse MI

**Figure 6:**
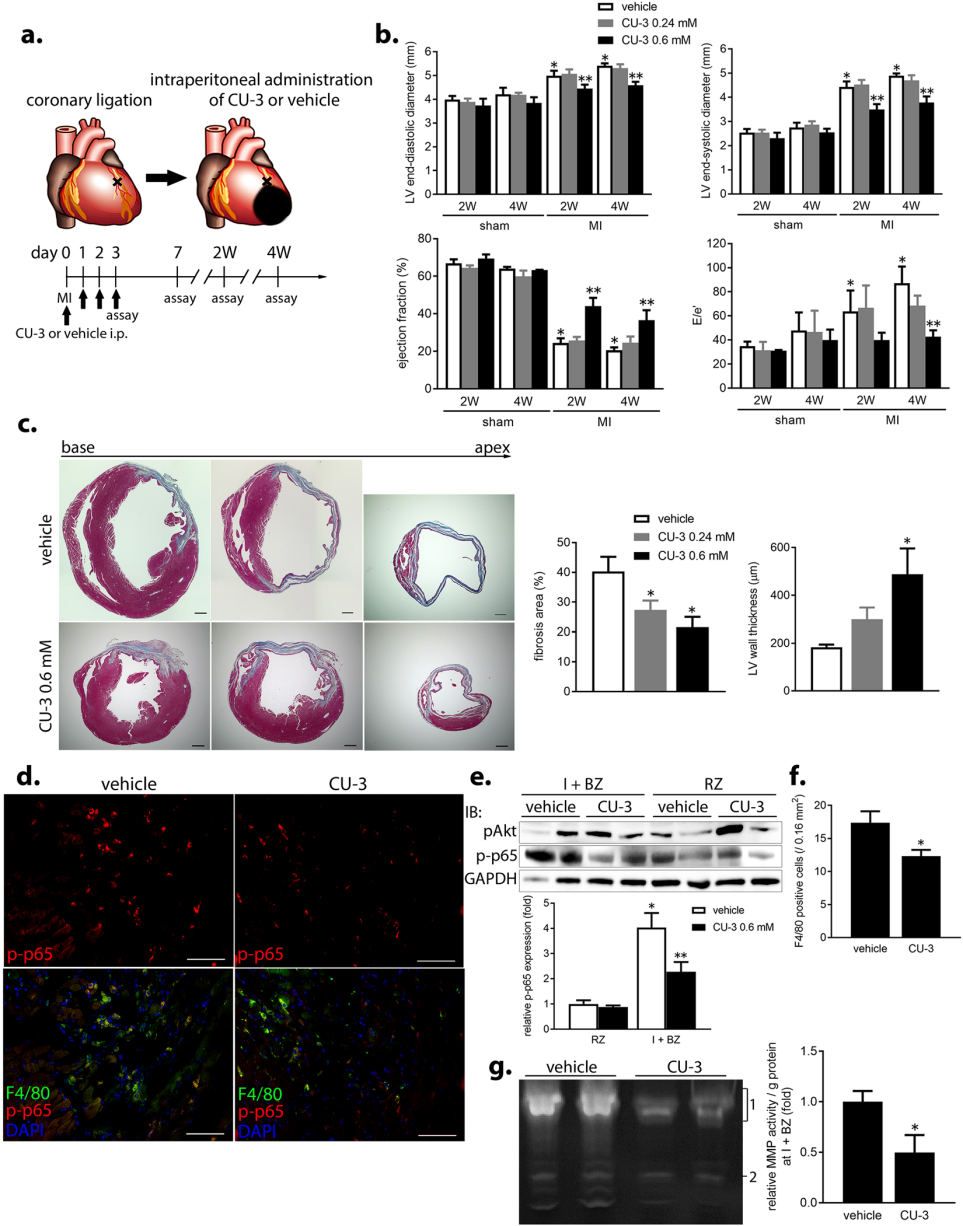
Short-term, systemic delivery of a selective DGKα inhibitor mitigates acute inflammation and adverse LV remodeling in mouse MI. (**a**) Schematic diagram showing systemic delivery of the selective DGKα inhibitor CU-3 or vehicle (administered intraperitoneally) for 4 days after coronary artery ligation. (**b**) Echocardiographic parameters at 2 and 4 weeks in the sham or MI groups treated with vehicle, low or high dose of CU-3. *p < 0.05, compared to the sham group; **p < 0.05, compared to the MI + vehicle group. (**c**) (left) Representative Masson’s trichrome staining at 4 weeks post-treatment. Scale bars, 500 μm. (right) Fibrosis area (%) and LV wall thickness in each MI group. *p < 0.05, compared to the MI + vehicle group. (**d**–**f**) Decrease in NFκB p65 activation and infiltration of F4/80-positive MQs in the I + BZs of the MI + CU-3 group at day 3 analyzed by immunofluorescent staining (**d**), western blot (**e**), and the number of F4/80-positive cells (**f**). Scale bars, 60 μm. *p < 0.05, compare to the RZ; **p < 0.05, compared to the MI + vehicle group (n = 5 per group). (**g**) Total MMP activity in the I + BZs at day 3. MMP9 and MMP2 are labeled 1 and 2, respectively.

As the CU-3-mediated inhibitory effects on AM function were the most remarkable *in vitro*, we focused on this inhibitor to evaluate the effects of systemic delivery on LV structure and function in mouse MI. CU-3 or vehicle (control) was administered intraperitoneally for 4 days after coronary artery ligation (Fig. 6a). At 2 weeks post-treatment, the highest dose of CU-3 (0.6 mM) effectively decreased LV end-diastolic and end-systolic diameters and increased ejection fraction (Fig. 6b) and global longitudinal strain (Supplementary Fig. S6a) compared to that in the control, without affecting systolic blood pressure, heart rate, and heart weight (Supplementary Fig. S6b). Furthermore, E/e’ was improved by 4 weeks post-treatment in the MI + CU-3 group (Fig. 6b), indicating amelioration of adverse LV remodeling in the chronic phase of MI. Survival rate was similar between treatment groups (Supplementary Fig. S6c). Histological analysis also revealed that fibrosis was limited to 53% and the LV wall was thicker at 4 weeks post-treatment in the MI + CU-3 group compared to that in the control group (Fig. 6c).

Focusing on the acute phase of MI at 3 days post-treatment, CU-3 administration significantly inhibited NFκB p65 activation and F4/80-positive MQ infiltration in the I + BZs (Fig. 6d–f). Moreover, total MMP activity and MMP9 protein expression were notably decreased in the I + BZs of the MI + CU-3 group (Fig. 6g and Supplementary Fig. S6d). In contrast, the extent of NFκB p65 activation and MMP activity in the I + BZs were similar in both groups at 7 days post-treatment (Supplementary Fig. S6e), indicating that CU-3 may only limit acute inflammation early on, but does not delay the overall repair process during MI. These data suggest that short-term, systemic delivery of a selective DGKα inhibitor reduces MQ infiltration, inflammation, and MMP activity during the acute phase and further mitigates adverse LV remodeling during the chronic phase in mouse MI.

## Discussion

In this study, we treated stressed cardiomyocytes with conditioned media derived from various cell types *in vitro* and identified AM as an Akt-activating hepatokine. Distribution of AM protein transiently increased in the I + BZs during the acute phase in mouse MI, reflecting infiltration of AM-bound MQs and interstitial AM accumulation. AM stimulation activated Akt, NFκB, and ERK1/2 signaling in addition to enhancing MQs migration and inflammation in CFBs and MQs, while inhibiting fibrogenesis-related mRNA expression *in vitro*. Intramyocardial administration of AM protein augmented MQ infiltration, inflammation, and MMP9 mRNA expression in the I + BZs, disturbed fibrotic repair, and drove cardiac rupture during the acute phase of MI. A protein-lipid overlay and lipid pull-down assays indicated that plasma membrane phospholipid PA partly mediates AM function. Indeed short-term, systemic delivery of CU-3, a selective inhibitor of DGKα-mediated PA biosynthesis, reduced MQ infiltration, inflammation, and MMP activity during the acute phase and mitigated LV remodeling during the chronic phase in MI.

AMBP is a glycoprotein proteolytically processed into AM and bikunin. Interestingly, administration of only AM, but not bikunin, showed sustained Akt activation from as early as 5 min in treated cardiomyocytes. Protein expression of AM transiently increased in the I + BZs at both the epicardial and endocardial sides beginning from day 1, peaking at day 3, and returning to baseline at day 7 during mouse MI. Notably, these changes occurred without any associated change in *AM* mRNA expression. Further, although AM protein was co-expressed with F4/80 and vimentin, mRNA expression of AM was largely undetected in cultured MQs even after lipopolysaccharide stimulation. Serum concentration of AM was also unchanged during both mouse and human MI compared to that in healthy controls, which is supported by observations in various diseases^15^. Therefore, it is reasonable to conclude that the observed increase in AM distribution mainly reflects infiltration of AM-bound MQs into the I + BZs during the acute phase of MI. Nevertheless, whether the binding ability of AM to MQs or to circulating monocytes increases during MI needs further investigation.

While *AM* mRNA expression was largely unaffected in cardiomyocytes, AM administration in CFBs greatly increased the downstream mRNA expression of CCL2, proinflammatory cytokines, and MMPs especially IL-1β and MMP9 as well as secretion of these cytokines, while decreasing the expression of a myofibroblast marker αSMA and collagen 3a1 *in vitro*. Interestingly, AM stimulation also augmented the mRNA expression of a resident fibroblast marker platelet-derived growth factor receptor α^28^, implying that AM may regulate cardiac fibroblast transdifferentiation to myofibroblast. In MQs, AM stimulation increased the mRNA expression of iNOS and IL-6, whereas significantly inhibited markers for IL-13-induced polarization to the alternatively activated phenotype, such as arginase 1, IL-10, Fizz, and Mrc1. These data suggest that AM may even regulate MQ polarization to the alternatively activated phenotype. Moreover, AM augmented the mRNA expression of ICAM-1 and E-selectin, and directly promoted MQ migration. CCL2 and IL-1β secreted from CFBs promote MQ migration and activation to a more inflammatory phenotype, while the inflammatory MQs in turn promoted the CFBs to secrete more proinflammatory cytokines and MMPs. Thus, a vicious positive feedback loop is formed involving excessive MMP production, amplification of inflammation, and inhibition of fibrotic repair, a phenomenon that has been previously observed in MI^7,8^. In parallel to these *in vitro* effects, intramyocardial AM administration into the BZ significantly augmented MQ infiltration, Akt activation, and mRNA expression of MMP9 and iNOS at day 3, resulting in a 2.8-fold increase in death due to acute cardiac rupture compared to that in the control group. Moreover, the surviving mice tended to have more insufficient formation of collagen fibrils at 2 weeks post-treatment. Collectively, these *in vitro* and *in vivo* data strongly support the proinflammatory properties of AM in CFBs and MQs during MI.

Although the shotgun proteomic analysis could not find the membrane functional receptor of AM, a protein-lipid overlay assay and lipid pull-down assay led us to the discovery of an AM-PA interaction. AM is a heme-binding protein of the lipocalin family which has a biophysical or biochemical retentive mechanism that can trap the small hydrophobic compounds including lipids by the lipocalin pocket^14^. On the other hand, a negatively charged PA could flip to the outer leaflet of the membrane bilayer when the charge is neutralized^29^. Therefore, it is considered possible that exogenous AM protein biophysically or biochemically binds to PA in the plasma membrane in certain circumstances. Indeed a lipocalin homolog von Ebner’s-gland protein has been shown to bind phospholipids^30^. Nevertheless, the mechanism of AM binding to PA needs further investigation, including mapping the PA binding sites in AM and generating PA binding mutants to assess the regulatory function.

As to AM binding to PI(4)P, AM-binding PA can promote the affinity of PI(4)P 5-kinase for its substrate PI(4)P to produce phosphatidylinositol (4, 5)-diphosphate^31^. Phosphatidylinositol (4, 5)-diphosphate then regulates migration, adhesion, phagocytosis, secretion, and gene transcription in various immune cells as a substrate of phospholipase C and PI 3-kinase^32^. Therefore, it would be possible for AM, PA, and PI(4)P to form a complex and that PI(4)P works in PA downstream signaling pathway.

PA is a signaling phospholipid that mediates a variety of biological function, including signal transduction via protein kinase C, Ras, Akt, mammalian target of rapamycin, NFκB, phosphatidylinositol-4-phosphate 5-kinase, and sphingosine kinase 1^31,33–35^, cell migration, actin-myosin cytoskeletal reorganization, and membrane vesicle trafficking of endocytosis and exocytosis^36^. Based on these function, PA is crucial in the progression of an assortment of immune disorders, thrombosis, diabetes mellitus, neurodegeneration, and cancer metastasis^34–37^, which may be the case in post MI remodeling. PA is synthesized via three pathways: from phosphatidylcholine by phospholipase D; from diacylglycerol by DGK; or from lysophosphatidic acid by AGPAT^23,24^. There are 10 isoforms of DGK in mammals^38^, and DGK isoform-specific expression and function is apparent in immune cells and cardiac function^39,40^. In line with this, various inhibitors of PA synthesis have been developed, including the phospholipase D1/D2 inhibitor FIPI and the non-selective DGK inhibitor R59949, etc. Further, a small chemical compound CU-3 was recently identified by high-throughput screening as a highly selective DGKα inhibitor that is more potent than the other DGK inhibitors^27^. To determine if PA actually mediates AM function, we analyzed the downstream effects of various PA synthesis inhibitors. Indeed the inhibitory effects on AM signaling and function was evident in CFBs and MQs after treatment with the DGK inhibitors.

While this study highlights a novel aspect of cardio-hepatic interaction in MI, there are some limitations. First, the cardiac effects and effective time window of AM may differ between our mouse MI model (which uses permanent coronary artery ligation) and other ischemia-reperfusion injury model that involves the partial survival of cardiomyocytes. Second, loss-of-function studies of AM were not presented in this research because we have created *AM* knockout mice, but could not obtain *AM*^−/−^ mice probably due to embryonic lethality. Furthermore, a neutralizing antibody to AM is not commercially available at present. Third, changes in the PA amount within the plasma membrane were not quantitated *in vitro* and *in vivo* as it is difficult to measure accurately and reliably with the available technology. As the DGKα inhibitor used could affect not only macrophages but also cardiac fibroblasts, cardiomyocytes, vascular endothelial cells, or vascular smooth muscle cells, our data presented here is not sufficient to conclude that macrophages are the central mediator for AM effect in MI. However, while additional research is warranted, the current study provides a solid foundation for the continued investigation of AM in cardiovascular disease.

In conclusion, the discoveries presented here concerning the underlying mechanism of proinflammatory hepatokine AM introduce a novel pharmacological option for post MI remodeling and heart failure. AM administration triggered acute cardiac rupture in mouse MI, while an inhibitor to block AM-PA interaction was shown to mitigate acute inflammation and adverse LV remodeling after MI, a major burden for acute MI patients. Our findings not only elucidate a previously unknown aspect of cardio-hepatic interaction during cardiovascular diseases, but could also be expanded to other systemic inflammatory diseases, including autoimmune diseases, steatohepatitis, diabetes mellitus, and neurodegenerative diseases. While additional research is needed, this study represents the first step towards developing a novel pharmacological therapy that exploits the role of AM signaling in disease.

## Methods

### Animals

Male mice in a C57BL/6J background were purchased from Japan CLEA (Tokyo, Japan). Mice were kept in a temperature-controlled room with a 14:10 hrs of light:dark cycle in specific pathogen-free conditions at the Institute of Laboratory Animals of Graduate School of Medicine in Kyoto University. All protocols of animal study in this research were approved by Animal Experimentation Committee of the Kyoto University and conformed to the NIH *Guidelines for the Care and Use of Laboratory Animals* and ARRIVE (Animal Research: Reporting of *In Vivo* Experiments) guidelines.

### *In vitro* screening of the conditioned media

L6 rat skeletal myoblasts (CRL-1458; ATCC, Manassas, USA), Huh7 human hepatoma cells (JCRB0403; JCRB Cell Bank, Osaka, Japan), MES13 mouse renal mesangial cells (CRL-1927; ATCC), primary human coronary artery endothelial cells (C-12221; PromoCell, Heidelberg, Germany), primary rat aortic smooth muscle cells, and primary rat hepatocytes were plated each on a 100 mm dish. These cells were incubated at 37 °C in DMEM containing 1% glucose (Nacalai Tesque, Kyoto, Japan) supplemented with 10% FBS, antibiotics (Gibco, Waltham, USA), and non-essential amino acids (Gibco) to become confluent, and serum-starved for 24 hrs. Each conditioned medium was collected and filtered by a cell strainer (Thermo Fischer Scientific, Waltham, USA). In parallel, primary neonatal rat cardiomyocytes were serum-starved for 24 hrs and subjected to 10^−6^ M doxorubicin (Sigma-Aldrich, St. Louis, USA) for 12 hours or 1% hypoxia by AnaeroPack Kenki (Mitsubishi Gas Chemical Company, Inc., Tokyo, Japan) for 9 hrs, with or without administration of the above conditioned medium. After stimulation, Akt and caspase-3 activation was analyzed in cell lysates of the cardiomyocytes by Western blot.

### Protein Purification

Protein purification was performed with AKTApurifier UPC 10 (GE Healthcare Life Sciences, Pittsburgh, USA), a fraction collector Frac-950, and UNICORN 5.20 software to fractionate Huh7-derived conditioned medium based on Akt-activating function on cardiomyocytes. All procedures were conducted at 4 °C. One hundred ml of the conditioned medium was precleared by centrifugation at 10,000 g for 10 min. It was subjected to intermediate purification by anion exchange chromatography of HiTrap Q HP column (GE Healthcare Life Sciences) with 20 mM Tris HCl, pH 7.4 at a flow rate of 2.5 ml/min. The sample was fractionated with a gradient of sodium chloride and the range of 0.1–0.3 M fractions was selected from the preliminary result. The selected fractions were further concentrated with Amicon Ultra-4, Ultracel, 10 kDa (Merck, Darmstadt, Germany) and removed phenol red with Bio-Spin 6 column (Bio-Rad, Hercules, USA). Next, albumin was depleted from these fractions with Albumin & IgG Depletion Spin Trap (GE Healthcare Life Sciences). The sample was further separated to 1 ml per fraction by gel filtration of 50 ml Superdex 200 10/300 GL column (GE Healthcare Life Sciences) with 20 mM sodium phosphate, pH 7.4 at a flow rate of 0.25 ml/min. The buffer in each fraction was exchanged with PD-10 column (GE Healthcare Life Sciences) for stimulation to cardiomyocytes.

### Protein identification by mass spectrometry

The selected fraction by gel filtration was electrophoresed in a 12% NuPAGE Bis-Tris Mini gel of SDS-polyacrylamide gel (Thermo Fischer Scientific) and stained with Coomassie Brilliant Blue using the SimplyBlue SafeStain (Thermo Fischer Scientific). The visualized band was excised with a clean, sharp blade, and subjected to mass spectrometry. For protein identification by peptide mass fingerprinting, excised protein spots were digested with trypsin (Promega, Madison, USA), mixed with α-cyano-4-hydroxycinnamic acid in 50% acetonitrile/0.1% TFA, and subjected to MALDI-TOF analysis (Microflex LRF 20; Bruker Daltonics, Billerica, USA). Spectra were collected from 300 shots per spectrum over m/z range 600–3000 and calibrated by two point internal calibration using Trypsin auto-digestion peaks (m/z 842.5099, 2211.1046). Peak list was generated using Flex Analysis 3.0. Threshold used for peak-picking was as follows: 500 for minimum resolution of monoisotopic mass, 5 for S/N. The search program MASCOT, developed by The Matrixscience (http://www.matrixscience.com/), was used for protein identification by peptide mass fingerprinting. The following parameters were used for the database search: trypsin as the cleaving enzyme, a maximum of one missed cleavage, iodoacetamide (Cys) as a complete modification, oxidation (Met) as a partial modification, monoisotopic masses, and a mass tolerance of ±0.1 Da. PMF acceptance criteria is probability scoring. All chemicals used in the mass spectrometry were of analytical grade. 4-Sulfophenyl isothiocyanate, a- cyano-4-hydroxycinnamicacid, sodium bicarbonate, and ammonium bicarbonate were purchased from Sigma-Aldrich (St. Louis, USA). The MALDI-TOF analysis was performed by outsourcing to GENOMINE, Inc. (Kyungbuk, Korea).

### Human study

Blood samples and clinical data were collected from the consecutive patients who admitted to our department with a diagnosis of acute MI and required emergent coronary angiography. Diagnosis of acute MI was made by at least one cardiologist, based on symptom, electrocardiography, laboratory tests, and echocardiography. The inclusion criteria was 20–85 years old, estimated glomerular filtration rate ≥55 ml/min/1.73 m^2^, and ST-elevation MI by complete occlusion of the main branch of either right coronary artery, left anterior descending coronary artery, or left circumflex coronary artery. Blood samples were collected within 24 hrs after the onset of acute MI in serum separator tubes (Neotube; Nipro, Osaka, Japan) and left at room temperature for at least 30 min. Then, the samples were centrifuged at 3500 rpm for 10 min and the serum was dispensed to 2 ml tubes as aliquots and stored at −80 °C until analysis. Clinical data were obtained from electronic medical records. The inclusion criteria of control subjects was asymptomatic individuals who received an annual medical check-up, no past history of cardiovascular diseases, and normal electrocardiographic finding. The criteria about age and renal function in control subjects was the same as that in acute MI patients. Serum concentration of AM was analyzed by latex agglutination turbidimetry (LX reagent ‘Eiken’ α_1_-M-III; Eiken Chemical Co., Tokyo, Japan).

The protocol of human study was approved by the Institutional Review Board of Kyoto University Hospital, and written informed consent was obtained from all participants prior to inclusion in the study. The study was carried out in accordance with the Declaration of Helsinki, and the participants were identified by number, not by name.

### Mouse MI model

Myocardial infarction was created by permanent ligation of the left anterior descending coronary artery in mouse as previously described^3^ with minor modifications. In brief, 9-week-old male mice were anesthetized intraperitoneally with pentobarbiturate sodium (Somnopentyl; Kyoritsuseiyaku, Tokyo, Japan). After intubation a mouse was set on a respirator (SN-480-7; Shinano Corporation, Tokyo, Japan), and left thoracotomy was performed to visualize the coronary artery under an optical microscope. Then the left anterior descending artery was ligated with a 7-0 polypropylene suture (Ethicon, Somerville, USA) at the level 1 mm below an inferior edge of the left atrial appendage. Successful ligation was confirmed by blanching of the myocardium. Sham-operated mice underwent the identical procedure without coronary artery ligation. Mice that died within 24 hrs after operation were excluded from the analysis. The deceased mice were subjected to an autopsy to determine the cause of death: cardiac rupture was judged by the presence of blood coagulation in the pericardium and the chest cavity.

In the experiment of intramyocardial administration, 20 μl of recombinant murine AM protein (50 μg/ml in PBS; MBS2012437, MyBioSource, San Diego, USA) or PBS was manually injected at three sites in the BZ by 0.1 ml Hamilton syringe (No. 710, Reno, USA) with 30 gauge needle (No. 90310) just after ligation by the previously described method^22^. Preservatives and endotoxins including lipopolysaccharide in the above recombinant murine AM protein were removed by diafiltration and buffer exchange to PBS with Amicon Ultra-0.5, Ultracel, 10 kDa (Merck) prior to injection. In a preliminary experiment to find retention period after injection, human AM protein was injected by the above method, and detected with anti-human AMBP antibody (sc-81948; Santa Cruz Biotechnology). In the experiment of systemic delivery of CU-3, 50 μl of low or high dose of CU-3 (0.24 mM or 0.6 mM in dimethyl sulfoxide, respectively; GLXC-07641, Glixx Laboratories, Hopkinton, USA) or vehicle (dimethyl sulfoxide; Sigma-Aldrich) was administered intraperitoneally for 4 days after ligation.

### Echocardiography and measurement of blood pressure and heart rate

Mouse cardiac function was assessed by the same tester using Vevo 2100 echocardiography (VisualSonics, Toronto, Canada) with a 18–38 MHz sector-array transducer at the indicated time points after sham or MI operation (n = 3 or 6 per group, respectively) as described previously^41^. Mice were anesthetized with 1–2% isoflurane inhalation for the heart rate to be kept at 480 ± 30 bpm during examination. LV wall thickness and diameters were measured with M mode of the parasternal short axis view and averaged from 3 beats. Mitral velocities of E and e’ were measured with pulsed-wave Doppler mode of the apical view. Global longitudinal strain by speckle tracking imaging was calculated by VevoStrain^TM^ according to the manufacturer’s instructions.

Blood pressure and heart rate were measured in conscious mice using the indirect tail-cuff method (BP-98AW ver. 2.12; Softron, Tokyo, Japan) at 2 and 4 weeks after MI (n = 4 per group).

### Shotgun proteomic analysis for screening AM-binding proteins

We used primary neonatal rat CFBs cultured on three 150 mm dishes at passage 3. After serum-starvation for 24 hrs, the cells were washed twice and medium was changed, and 10 μg/ml of native human AM protein (ab96149; Abcam, Cambridge, England) was added to the cells and incubated for 1 hr at 4 °C. For crosslinking cell surface proteins, 2 mM of water-soluble, membrane-impermeable DTSSP (Thermo Fischer Scientific) was added to the cells for 30 min at room temperature. Crosslinking was stopped by incubation with 20 mM Tris, pH 7.5 for 15 min. Plasma membrane proteins were then isolated with Minute Plasma Membrane Protein Isolation Kit (Invent Biotechnologies, Inc., Eden Prairie, USA) in PBS with 0.2% Triton X-100 (Wako Pure Chemical Industries, Osaka, Japan). Next, immunoprecipitation of isolated membrane proteins was performed with Immunoprecipitation Kit Dynabeads® Protein G (Thermo Fischer Scientific). Each 20 μg of proteins were incubated with either anti-human AM antibody (sc-135665; Santa Cruz Biotechnology, Dallas, USA) or control mouse IgG (BD Biosciences, San Jose, USA) for 1 hr at 4 °C, and eluted target proteins were shortly electrophoresed in a SDS-polyacrylamide gel and stained with the SimplyBlue SafeStain. The area from the well-bottom to the dye-front was excised out for each lane with a clean, sharp blade and subjected to mass spectrometry.

Mass spectrometry was conducted at the Medical Research Support Center in Kyoto University. The proteins in gel pieces were digested using In-gel Tryptic Digestion Kit for Mass Spectrometry (Thermo Fisher Scientific) according to the manufacturer’s instructions. The recovered peptides were resuspended in 0.1% formic acid and separated using nano-flow liquid chromatography (Nano-LC-Ultra 2Dplus System, Eksigent, Dublin, USA), which was used in a trap and elute mode with trap column (200 μm × 0.5 mm ChromXP C18-CL 3 μm 120 Å, Eksigent) and analytical column (75 μm × 15 cm ChromXP C18-CL 3 μm 120 Å, Eksigent). The separation was carried out using a binary gradient with solvent A (0.1% formic acid) and B (0.1% formic acid, 80% acetonitrile). The gradient program used was as follows: 2–40% B in 125 min, 40–90% B in 1 min, 90% B for 5 min, 90–2% B in 0.1 min, 2% B for 18.9 min, at 300 nL/min. The eluates from nano-LC were directly infused to the mass spectrometer (TripleTOF 5600+ system, SCIEX, Framingham, USA).

The datasets were acquired with the information-dependent acquisition method. The identification of peptides/proteins was carried out using ProteinPilot software version 4.5beta (SCIEX) with UniProtKB/Swiss-Prot database (Rattus norvegicus, June 2014) appended with known contaminant database (SCIEX). The relative abundances of the identified proteins were estimated through label-free quantification using Progenesis QI for Proteomics software (NSupplementaryar Dynamics, Newcastle upon Tyne, England). The peptide abundance was normalized to all proteins and the protein abundance was calculated through the relative quantification using non-conflicting peptides. The methods for normalization and relative quantification were provided by Progenesis QI for Proteomics software. Proteins identified by at least 2 distinct peptides having at least 95% confidence enriched in the AM-binding sample are shown in Supplementary Table S3.

### Protein-lipid overlay assay

Protein-lipid overlay assay was performed for membrane lipids and sphingolipids with Membrane Lipid Strips (P-6002; Echelon Biosciences, Salt Lake City, USA) and Sphingo Strips (S-6000; Echelon Biosciences), respectively according to the manufacturer’s instructions. In brief, the strip was incubated with the blocking buffer (PBS with 0.1% Tween-20 and 3% fatty acid-free BSA) for 1 hr at room temperature, and 1 μg/ml of recombinant murine AM protein (MBS2012437; MyBioSource) was added to the strip for 1 hr. Primary anti-mouse AM antibody (sc-366637; Santa Cruz Biotechnology), secondary HRP-conjugated anti-rabbit antibody, and Immobilon Western Chemiluminescent HRP Substrate (Merck Millipore, Billerica, USA) were used to detect positive spots. Signals were detected with ImageQuant LAS4000 Mini (GE Healthcare Life Sciences).

### Lipid-protein pull-down assay

Lipid-protein pull-down assay was performed with lipid coated beads (P-B000, P-B0PA, P-B004a, P-B0PE; Echelon Biosciences) as the manufacturer’s instructions. Briefly, 50 μl of the beads coated either with PA, PI(4)P, or phosphatidylethanolamine were incubated with 5 μg of recombinant murine AM protein (MyBioSource) in a binding buffer (10 mM HEPES, pH 7.4, 150 mM NaCl, and 0.5% Igepal (Sigma-Aldrich)) for 3 hrs at room temperature. The beads were then washed for 5 times, pelleted by centrifugation, and subjected to a western blot using anti-mouse AM antibody (sc-366637; Santa Cruz Biotechnology). The beads without lipid were used as a negative control.

### Cell isolation and culture

Neonatal rat cardiomyocytes and CFBs as well as adult rat hepatocytes and aortic smooth muscle cells were isolated as previously described^42,43^, with minor modifications. Cardiac cells were isolated from 2 day-old Wistar rats, and cardiomyocytes and CFBs were purified by Percoll density gradient centrifugation (Sigma-Aldrich). These cells and J774.1 mouse peritoneal MQs (JCRB0018; JCRB Cell Bank) were cultured in DMEM with 10% FBS, antibiotics, and non-essential amino acids.

For bone marrow-derived MQs, bone marrow cells were isolated from 12 week-old male WT mice as previously described^44^. The cells were then plated into 12-well plates at 1.5 × 10^6^ cells/well in 1 mL of bone marrow differentiation media (RPMI1640 (Gibco) supplemented with 10% FBS, 10% L929-conditioned media, 100 U/mL penicillin, 100 μg/mL streptomycin, and 2 mM L-glutamine). On day 3, extra 1 mL of differentiation media were added to the cells and incubated for another 3 days. Human coronary artery endothelial cells were cultured as previously described^4^. We used neonatal rat cardiomyocytes and adult rat hepatocytes without passage, and neonatal rat CFBs, adult rat aortic smooth muscle cells, and human coronary artery endothelial cells at passage 3 to 4 for experiments.

### Preparation and transfection of siRNAs and plasmids

For knockdown of rat annexin A2 or AGPAT1/2, duplex oligoribonucleotides were designed using Stealth RNAi^TM^ (Thermo Fischer Scientific) or MISSION® siRNA (Sigma-Aldrich), respectively. The sense strand of siRNA oligoribonucleotides were as follows: ACUUCGACGCUGAGAGGGAUGCUUU for siannexin A2, CACAGGAGACGCUAUCAGUTT for siAGPAT1, GAUUGCCAAGCGUGAGCUATT for siAGPAT2. The siRNA negative control was Stealth RNAi^TM^ siRNA negative control med GC (12935-300; Thermo Fischer Scientific) or MISSION siRNA Universal Negative Control (SIC-001; Sigma-Aldrich), respectively.

For overexpression of rat annexin A2, its cDNA was first obtained by RT-PCR of rat CFBs RNA using Verso cDNA Synthesis Kit (Thermo Fischer Scientific) and iProof High-Fidelity DNA Polymerase (Bio-Rad). Then annexin A2 cDNA was inserted into pcDNA3.1 (+) plasmid vector (Thermo Fischer Scientific) with NheI-HF enzyme (New England BioLabs, Ipswich, USA) and DNA Ligation Kit <Mighty Mix> (Takara Biotechnologies, Shiga, Japan). The plasmid vector for transfection was prepared with ECOS Competent *E*.*coli* JM109 (NIPPON GENE, Toyama, Japan) and Genopure Plasmid Midi Kit (Roche Diagnostics, Basel, Switzerland). The negative control was empty plasmid vector.

The plasmid vectors or siRNA oligoribonucleotides (30 nM each) were transfected to primary rat CFBs with Lipofectamine^TM^ 3000 Reagent (Thermo Fisher Scientific) and Opti-MEM Reduced Serum Medium according to the manufacturer’s instructions. For co-transfection of siRNAs against AGPAT1 and AGPAT2, 20 nM of each siRNA was used. The transfection efficiency of BLOCK-iT^TM^ Alexa Fluor Red Fluorescent Control to CFBs was more than 80% at 48 hrs. After 48 hrs of transfection, the CFBs was serum-starved for 24 hrs and stimulated with 20 μg/ml of AM protein.

### Quantitative PCR

Native human AM (ab96149; Abcam) or bikunin (MBS142876; MyBioSource) protein was administered to rat or mouse cells *in vitro*. In J774 MQs with IL-13 stimulation, the cells were serum-starved for 24 hrs and polarized to the alternatively activated phenotype by stimulation with 50 ng/ml recombinant human IL-13 (SRP3274, animal component-free, Sigma-Aldrich) for 24 hrs. Then the cells were stimulated with AM protein under IL-13 for the indicated time points. In some experiments using the inhibitors of PA synthesis *in vitro*, dimethyl sulfoxide (control, final concentration of 0.1%), 1 μM FIPI hydrochloride (F5807; Sigma-Aldrich), 40 μM R59949 (D5794; Sigma-Aldrich), or 5 μM CU-3 was administered to the cells 20 min prior to 20 μg/ml AM stimulation. Monolayer cells were rinsed twice with serum-free medium and serum-starved for 24 hrs before stimulation, and lysed directly on culture dishes, and tissue samples were homogenized with a polytron homogenizer in 1 ml of TriPure Isolation Reagent (Roche Diagnostics). Total RNA was extracted according to the manufacturer’s instructions. For quantitative PCR analysis, single-strand cDNA was synthesized from RNA with Verso cDNA Synthesis Kit, and quantitative PCR was performed with THUNDERBIRD SYBR qPCR Mix (TOYOBO, Osaka, Japan) and StepOnePlus Real-Time PCR System with StepOne Software ver.2.3 (Applied Biosystems, Foster City, USA). Relative expression levels were normalized to β-actin or GAPDH (n = 4~5 per group). The primer sequences used are listed in Supplementary Table S4.

### Western blot analysis

Western blot analysis was performed by standard procedures as described previously^41^. In some experiments, 10 μM LY294002 (Merck Millipore) was administered to the cells 20 min prior to stimulation with human AM protein (ab96149; Abcam). Cell lysates were prepared in chilled RIPA lysis buffer with Complete Mini Protease Inhibitor Cocktail (Roche Diagnostics), 1 mmol/L sodium fluoride (Sigma-Aldrich), 1 mmol/L sodium orthovanadate (Sigma-Aldrich), and 1 mmol/L PMSF (Sigma-Aldrich). Tissue samples were homogenized with a polytron homogenizer and lysed in T-PER Tissue Protein Extraction Reagent (Thermo Fisher Scientific) with Protease Inhibitor Cocktail (Sigma-Aldrich). All samples were run on SDS-PAGE with 4–12% NuPAGE Bis-Tris Mini gels and transferred to a Protran nitrocellulose transfer membrane (GE Healthcare Life Sciences). The membrane was blocked with Blocking One or Blocking One P (Nacalai Tesque) for 1 hr, incubated with the primary antibody overnight at 4 °C followed by the secondary antibody for 1 hr. The membrane was then detected with Immobilon Western Chemiluminescent HRP Substrate or Pierce ECL Western Blotting Substrate (Thermo Fisher Scientific) in ImageQuant LAS4000 Mini. Densitometric analyses were performed with ImageJ ver.1.48 software (National Institute of Health, Bethesda, USA).

The primary antibodies used were: anti-phospho Akt (Ser473) (9271; Cell Signaling Technology, Danvers, USA); anti-total Akt (9272; Cell Signaling Technology); anti-caspase-3 (sc-7148; Santa Cruz Biotechnology); anti-human AMBP (sc-81948); anti-mouse AM (sc-366637); anti-phospho NFκB p65 (Ser536) (3033; Cell Signaling Technology); anti-NFκB2 p100/p52 (4882; Cell Signaling Technology); anti-phospho Stat3 (Ser727) (9134; Cell Signaling Technology); anti-phospho ERK1/2 (9101; Cell Signaling Technology), anti-annexin A2 (8235; Cell 0S0ignaling Technology); and anti-vimentin (sc-32322; Santa Cruz Biotechnology). HRP-conjugated anti-rabbit IgG or anti-mouse IgG (GE Healthcare Life Sciences) antibody was used as the secondary antibody. The amount of total protein was assessed by reblotting with anti-GAPDH antibody (2118; Cell Signaling Technology).

### Histology and immunostaining

Histological analysis and immunostaining were performed as previously described^41^. Mice were sacrificed with an overdose of anesthetics, and specimens were perfused with 4% paraformaldehyde (Wako Pure Chemicals), fixed overnight at 4 °C, and embedded in paraffin. For frozen sections, samples were embedded in OCT compound (Sakura Finetek, Tokyo, Japan) after fixation and frozen in liquid nitrogen. Masson’s trichrome staining was performed with Accustain Trichrome Stain (Masson) Kit (HT15; Sigma-Aldrich) after the sections were deparaffinized. For analysis of fibrosis area, the LV was sequentially sectioned at a 400 μm interval from a point of coronary artery ligation to the apex (10–12 sections in average per LV), and percentage of fibrosis area was calculated with ImageJ software in each section with Masson’s trichrome stain and averaged for analysis (n = 5 per group). LV wall thickness was measured as the thickness of the thinnest part in the fibrosis area (n = 5 per group).

For immunostaining of AM on paraffin-embedded sections, antigen retrieval was performed with HistoVT One (Nakalai Tesque, Kyoto, Japan) at 90 °C for 20 min, followed by blocking with Blocking One Histo (Nakalai Tesque) at room temperature for 10 min. The primary and secondary antibodies were then diluted in this blocking buffer. For another immunostaining, antigen retrieval was performed by heating in a microwave oven with 10 mM citrate buffer, pH 6.0 for 3 min. the sections were blocked with 5% donkey serum and incubated with the primary antibodies overnight at 4 °C, and then incubated with Alexa Fluor 488- or 594-conjugated secondary antibodies (Thermo Fisher Scientific and Jakson ImmunoResearch, West Grove, USA) with DAPI (Thermo Fisher Scientific). The slides were mounted in VECTASHIELD Mounting Medium (Vector Laboratories, Burlingame, USA), and observed under an immunofluorescence microscope (BIOREVO BZ-9000; Keyence, Osaka, Japan). The number of F4/80 positive cells in the BZ was counted in more than 12 random fields of the frozen sections and averaged.

The primary antibodies used were: anti-mouse AM (sc-366637; Santa Cruz Biotechnology for paraffin-embedded sections and LS-C292739; LifeSpan Biosciences, Seattle, USA for frozen sections); anti-human AMBP (sc-81948); anti-F4/80 (clone CI:A3-1, MCA497RT; Bio-Rad); anti-vimentin (GP53; PROGEN Biotechnik, Heidelberg, Germany); anti-LY-6G (clone 1A8, 127601; Biolegend); anti-cardiac Troponin I (ab47003; Abcam); anti-phospho NFκB p65 (Ser536) (3033), and anti-MMP9 (sc-6840; Santa Cruz Biotechnology).

### Gelatin zymography

Each sample of 40 μg protein at I + BZs (n = 4~5 per group) was run on SDS-PAGE with Novex 10% Gelatin Zymogram Gels (Thermo Fisher Scientific) without heating or reducing according to the manufacturer’s instructions. After electrophoresis, the gel was renatured and developed for 14 hrs at 37 °C. The gel was then stained with the SimplyBlue SafeStain, and the clear bands were detected with ImageQuant LAS3000 Mini and analyzed with ImageJ software.

### Enzyme-linked immunosorbent assay

Blood samples were collected from the inferior vena cava in mouse and left at room temperature for 2 hrs. Then, the samples were centrifuged at 3500 rpm for 10 min and the serum was stored at −80 °C until analysis. Serum concentration of AM was examined in sham or MI with Mouse α1-Microglobulin ELISA Kit (MBS2505957, MyBioSource) according to the manufacturer’s instructions. In cultured CFBs, the cells were serum-starved for 24hrs and the medium was changed to the fresh one. The cells were incubated for 48hrs with or without 20 μg/ml of human AM protein stimulation to collect the conditioned medium. Concentration of TNFα and IL-1β in the conditioned medium was analyzed with Quantikine ELISA Kits for rat TNFα (RTA00, R&D Systems, Minneapolis, USA) and rat IL-1β (RLB00), respectively.

### MQ migration assay

J774 MQ migration was examined with CytoSelect 96-Well Cell Migration Assay (5 μm, Fluorometric Format) (Cell Biolabs, San Diego, USA) according to the manufacturer’s instructions. MQs were serum-starved for 24 hrs and plated on the upper chambers at a cell density of 2 × 10^5^ cells/well (4 wells per group). CFBs were incubated for 48 hrs with or without human AM protein (ab96149; Abcam) stimulation to collect the conditioned medium. After 18 hrs, migratory cells through the 5 μm pore were analyzed with 2030 ARVO X4 at 480 nm/520 nm.

### Tube formation assay

Tube formation assay of human umbilical vein endothelial cells (C-12200; PromoCell) was performed as previously described^41^, with minor modifications. After serum-starvation for 8 hrs, the cells were stimulated with or without 20 μg/ml of human AM protein (ab96149; Abcam) for 18 hrs and plated on a 24 well plate. Total tube length for 6 hrs in each well was observed with a microscope BZ-9000 (4 wells per group).

### Statistics

The measurements are presented as mean ± standard error of the mean except clinical characteristics of human study. For statistical comparisons, unpaired Student’s t-test (two groups, parametric), Mann-Whitney test (two groups, non-parametric), or one-way ANOVA with Sidak’s post-hoc test (three or more groups) were used. A p value of <0.05 was considered as statistically significant. Statistical analyses were performed with GraphPad Prism 7 (GraphPad Software, Inc., La Jolla, USA).

## Electronic supplementary material

Supplementary Information

## Data Availability

All data generated or analyzed during this study are included in this article (and its Supplementary Information files).

## References

[1] Nabel EG, Braunwald E (2012). A tale of coronary artery disease and myocardial infarction. N Engl J Med..

[2] Wilkins, E. *et al*. European Cardiovascular Disease Statistics. *European Heart Network*, Brussels, Belgium, http://www.ehnheart.org/cvd-statistics.html. AccessedJuly 6, 2017 (2017).

[3] Anzai A (2012). Regulatory role of dendritic cells in postinfarction healing and left ventricular remodeling. Circulation..

[4] Hilgendorf I (2014). Ly-6Chigh monocytes depend on Nr4a1 to balance both inflammatory and reparative phases in the infarcted myocardium. Circ Res..

[5] Lavine KJ (2014). Distinct macrophage lineages contribute to disparate patterns of cardiac recovery and remodeling in the neonatal and adult heart. Proc Natl Acad Sci USA.

[6] Nahrendorf M (2007). The healing myocardium sequentially mobilizes two monocyte subsets with divergent and complementary functions. J Exp Med..

[7] Prabhu SD, Frangogiannis NG (2016). The Biological Basis for Cardiac Repair After Myocardial Infarction: From Inflammation to Fibrosis. Circ Res..

[8] Frieler RA, Mortensen RM (2015). Immune cell and other noncardiomyocyte regulation of cardiac hypertrophy and remodeling. Circulation..

[9] Pavo N (2017). Sequential activation of different pathway networks in ischemia-affected and non-affected myocardium, inducing intrinsic remote conditioning to prevent left ventricular remodeling. Sci Rep..

[10] Bang C (2015). Intercellular communication lessons in heart failure. Eur J Heart Fail..

[11] Pal D (2012). Fetuin-A acts as an endogenous ligand of TLR4 to promote lipid-induced insulin resistance. Nat Med..

[12] Kharitonenkov A (2005). FGF-21 as a novel metabolic regulator. J Clin Invest..

[13] Misu H (2010). A liver-derived secretory protein, selenoprotein P, causes insulin resistance. Cell Metab..

[14] Akerstrom B, Gram M (2014). A1M, an extravascular tissue cleaning and housekeeping protein. Free Radic Biol Med..

[15] Akerstrom B, Logdberg L, Berggard T, Osmark P, Lindqvist A (2000). alpha(1)-Microglobulin: a yellow-brown lipocalin. Biochim Biophys Acta.

[16] Olsson MG (2011). Up-regulation of A1M/alpha1-microglobulin in skin by heme and reactive oxygen species gives protection from oxidative damage. PloS one.

[17] Olsson MG, Olofsson T, Tapper H, Akerstrom B (2008). The lipocalin alpha1-microglobulin protects erythroid K562 cells against oxidative damage induced by heme and reactive oxygen species. Free Radic Res..

[18] Sverrisson, K. *et al*. Extracellular fetal hemoglobin induces increases in glomerular permeability: inhibition with alpha1-microglobulin and tempol. *Am J Physiol Renal Physiol*. **306**, F442–448 (2014). 10.1152/ajprenal.00502.2013 24338823

[19] Wester-Rosenlof L (2014). A1M/alpha1-microglobulin protects from heme-induced placental and renal damage in a pregnant sheep model of preeclampsia. PloS one.

[20] Zager RA, Johnson AC, Frostad K (2016). An evaluation of the antioxidant protein alpha1-microglobulin as a renal tubular cytoprotectant. Am J Physiol Renal Physiol..

[21] Fleming TJ, Fleming ML, Malek TR (1993). Selective expression of Ly-6G on myeloid lineage cells in mouse bone marrow. RB6-8C5 mAb to granulocyte-differentiation antigen (Gr-1) detects members of the Ly-6 family. J Immunol..

[22] Lorchner H (2015). Myocardial healing requires Reg3beta-dependent accumulation of macrophages in the ischemic heart. Nat Med..

[23] Baldanzi G, Bettio V, Malacarne V, Graziani A (2016). Diacylglycerol Kinases: Shaping Diacylglycerol and Phosphatidic Acid Gradients to Control Cell Polarity. Front Cell Dev Biol..

[24] Nelson RK, Frohman MA (2015). Physiological and pathophysiological roles for phospholipase D. J Lipid Res..

[25] Su W (2009). 5-Fluoro-2-indolyl des-chlorohalopemide (FIPI), a phospholipase D pharmacological inhibitor that alters cell spreading and inhibits chemotaxis. Mol Pharmacol..

[26] Sato M (2013). Evaluations of the selectivities of the diacylglycerol kinase inhibitors R59022 and R59949 among diacylglycerol kinase isozymes using a new non-radioactive assay method. Pharmacology.

[27] Liu K (2016). A novel diacylglycerol kinase alpha-selective inhibitor, CU-3, induces cancer cell apoptosis and enhances immune response. J Lipid Res..

[28] Smith CL, Baek ST, Sung CY, Tallquist MD (2011). Epicardial-derived cell epithelial-to-mesenchymal transition and fate specification require PDGF receptor signaling. Circ Res..

[29] van Meer G, Voelker DR, Feigenson GW (2008). Membrane lipids: where they are and how they behave. Nat Rev Mol Cell Biol..

[30] Glasgow B, Abduragimov A, Farahbaksh Z, Faull K, Hubbell W (1995). Tear lipocalins bind a broad array of lipid ligands. Curr Eye Res..

[31] Jarquin-Pardo M, Fitzpatrick A, Galiano FJ, First EA, Davis JN (2007). Phosphatidic acid regulates the affinity of the murine phosphatidylinositol 4-phosphate 5-kinase-Ibeta for phosphatidylinositol-4-phosphate. J Cell Biochem..

[32] Tuosto L, Capuano C, Muscolini M, Santoni A, Galandrini R (2015). The multifaceted role of PIP2 in leukocyte biology. Cell Mol Life Sci..

[33] Delon C (2004). Sphingosine kinase 1 is an intracellular effector of phosphatidic acid. J Biol Chem..

[34] Foster DA (2009). Phosphatidic acid signaling to mTOR: signals for the survival of human cancer cells. Biochim Biophys Acta.

[35] Zhao C, Du G, Skowronek K, Frohman MA, Bar-Sagi D (2007). Phospholipase D2-generated phosphatidic acid couples EGFR stimulation to Ras activation by Sos. Nat Cell Biol..

[36] Nishikimi A (2009). Sequential regulation of DOCK2 dynamics by two phospholipids during neutrophil chemotaxis. Science..

[37] Elvers M., Stegner D., Hagedorn I., Kleinschnitz C., Braun A., Kuijpers M. E. J., Boesl M., Chen Q., Heemskerk J. W. M., Stoll G., Frohman M. A., Nieswandt B. (2010). Impaired  IIb 3 Integrin Activation and Shear-Dependent Thrombus Formation in Mice Lacking Phospholipase D1. Science Signaling.

[38] Topham MK, Epand RM (2009). Mammalian diacylglycerol kinases: molecular interactions and biological functions of selected isoforms. Biochim Biophys Acta.

[39] Takeishi Y, Goto K, Kubota I (2007). Role of diacylglycerol kinase in cellular regulatory processes: a new regulator for cardiomyocyte hypertrophy. Pharmacol Ther..

[40] Zhong XP, Guo R, Zhou H, Liu C, Wan CK (2008). Diacylglycerol kinases in immune cell function and self-tolerance. Immunol Rev..

[41] Hakuno Daihiko, Kimura Naritaka, Yoshioka Masatoyo, Mukai Makio, Kimura Tokuhiro, Okada Yasunori, Yozu Ryohei, Shukunami Chisa, Hiraki Yuji, Kudo Akira, Ogawa Satoshi, Fukuda Keiichi (2010). Periostin advances atherosclerotic and rheumatic cardiac valve degeneration by inducing angiogenesis and MMP production in humans and rodents. Journal of Clinical Investigation.

[42] Sanada S (2007). IL-33 and ST2 comprise a critical biomechanically induced and cardioprotective signaling system. J Clin Invest..

[43] Tomita K (2004). Pioglitazone prevents alcohol-induced fatty liver in rats through up-regulation of c-Met. Gastroenterology.

[44] Weischenfeldt J., Porse B. (2008). Bone Marrow-Derived Macrophages (BMM): Isolation and Applications. Cold Spring Harbor Protocols.

