# Supplementary material for: Hepatokine α1-Microglobulin Signaling Exacerbates Inflammation and Disturbs Fibrotic Repair in Mouse Myocardial Infarction
Source: Sci Rep. 2018 Nov 13;8:16749. doi: 10.1038/s41598-018-35194-w (PMC6233179; doi:10.1038/s41598-018-35194-w)

Supplementary Information

Hepatokine α1-Microglobulin Signaling Exacerbates Inflammation and Disturbs Fibrotic Repair in Mouse Myocardial Infarction

Daihiko Hakuno[1]*, Masahiro Kimura[1], Shinji Ito[2], Junko Satoh[2], Yasuhiro Nakashima[1], Takahiro Horie[1], Yasuhide Kuwabara[1], Masataka Nishiga[1], Yuya Ide[1], Osamu Baba[1], Hitoo Nishi[1], Tetsushi Nakao[1], Tomohiro Nishino[1], Fumiko Nakazeki[1], Satoshi Koyama[1], Ritsuko Hanada[1], Ruiz R. Randolph[1], Jin Endo[3], Takeshi Kimura[1], Koh Ono[1]

Author Affiliations:

[1] Department of Cardiovascular Medicine, Graduate School of Medicine, Kyoto University, 54 Kawaharacho, Shogoin, Sakyo-ku, Kyoto, 606-8507, Japan.

[2] Medical Research Support Center, Graduate School of Medicine, Kyoto University, 54 Kawaharacho, Shogoin, Sakyo-ku, Kyoto, 606-8507, Japan

[3] Cardiovascular Division, Department of Internal Medicine, Keio University School of Medicine, 35 Shinanomachi, Shinjuku-ku, Tokyo, Japan.

Supplementary Table S1. Raw data with peptide mass fingerprinting of fraction 3 in gel filtration

Accession number	Protein name	Score*	Expect	Number of masses matched
gi|4502067	AMBP	112	1.6e-06	10
gi|223373	complex-forming glycoprotein HC	96	5.5e-05	8
gi|374977533	Chain A, Crystal Structure of alpha-1-microglobulin	94	9.4e-05	8
gi|119584203	fibrinogen-like 1, isoform CRA_a	46	6.4	4
gi|146330428	immunoglobulin heavy chain variable region	42	15	3
gi|119584206	fibrinogen-like 1, isoform CRA_d	42	16	4
gi|119611487	hCG2039058	40	23	3
gi|67515417	fibrinogen-like 1	38	35	4
gi|16444966	HP-041	38	35	4
gi|22023090	LFIRE1	38	35	4
gi|42544189	fibrinogen-like protein 1 precursor	38	35	4
gi|211830458	TP53BP2	37	48	6

*Protein scores greater than 66 are significant ($p<0.05$).

For AMBP, protein sequence coverage: 39%, number of masses searched/matched: 19/10

Matched peptides are shown in red in AMBP protein sequence.

1 MRSLGALLLL LSACLAVSAG PVPTPPDNIQ VQENFNISRI YGKWYNLAIG

51 STCPWLKKIM DRMTVSTLVL GEGATEAEIS MTSTRWRKGV CEETSGAYEK

101 TDTDGKFLYH KSKWNITMES YVVHTNYDEY AIFLTKKFSR HHGPTITAKL

151 YGRAPQLRET LLQDFRVVAQ GVGIPEDSIF TMADRGECVP GEQEPEPILI

201 PRVRRAVLPQ EEEGSGGGQL VTEVTKKEDS CQLGYSAGPC MGMTSRYFYN

251 GTSMACETFQ YGGCMGNGNN FVTEKECLQT CRTVAACNLP IVRGPCRAFI

301 QLWAFDAVKG KCVLFPYGGC QGNGNKFYSE KECREYCGVP GDGDEELLRF

351 SN

Supplementary Table S2. Clinical characteristics of human control subjects and acute MI patients in this study

	control (n = 33)	acute MI (n = 37)	p value
age	56.0 ± 12.9	61.7 ± 12.0	0.06
sex (% male)	82	89	0.39
BMI	23.7 ± 3.1	25.9 ± 3.9	0.02
estimated GFR	73.8 ± 6.8	81.8 ± 14.1	0.02
diabetes mellitus (%)	3.0	32.4	0.001
hypertension (%)	24.2	51.4	0.02
infarcted branch (RCA: LAD: LCX)		19: 14: 4	

BMI, body mass index; GFR, glomerular filtration ratio; RCA, right coronary artery; LAD; left anterior descending coronary artery; LCX, left circumflex coronary artery. The measurements are presented as mean ± standard deviation.

Supplementary Table S3. Shotgun proteomic analysis of stimulated primary rat CFBs

Max fold change	Normalized abundance		Genes	Accession number
	AM	PBS		
83.47	250035.99	2995.43	Alpha-actinin-4	sp|Q9QXQ0|ACTN4
50.73	5902.07	116.34	Annexin A2	sp|Q07936|ANXA2
24.30	17014.20	700.24	Heat shock cognate 71 kDa protein	sp|P63018|HSP7C
8.45	2848.19	337.02	Glial fibrillary acidic protein	sp|P47819|GFAP
3.45	110360.26	32005.78	Vimentin	sp|P31000|VIME
3.12	31986.74	10267.54	Myosin-9	sp|Q62812|MYH9
2.46	14994.00	6085.56	Myosin-10	sp|Q9JLT0|MYH10
1.76	3711.49	2109.96	Tubulin beta-5 chain	sp|P69897|TBB5
1.52	36513.90	23946.72	Ig gamma-2C chain C region	sp|P20762|IGG2C
1.35	6017.92	4472.67	78 kDa glucose-regulated protein	sp|P06761|GRP78
1.25	2902.98	2328.77	Alpha-actinin-1	sp|Q9Z1P2|ACTN1
1.20	25842.57	21557.68	Actin, aortic smooth muscle	sp|P62738|ACTA
1.13	184196.83	163446.59	Ig gamma-2B chain C region	sp|P20761|IGG2B

Proteins identified by at least 2 distinct peptides having at least 95% confidence are shown.

Supplementary Table S4. PCR primer sequences used in this study

Species	Gene		Sequence
Mouse	*AM*	(F)	GAAACAGAGATCAGCATGACCAGTA
		(R)	CATTTGGATTTGTGGTAGAGGAACT
	ICAM-1	(F)	TCACCAGGAATGTGTACCTGAC
		(R)	GGCTTGTCCCTTGAGTTTTATG
	VCAM-1	(F)	TGACAAGTCCCCATCGTTGA
		(R)	ACCTCGCGACGGCATAATT
	L-selectin	(F)	ACCCACTCTCTTGGAGCTGA
		(R)	CAGGTTGGGCAAGTTAAGGA
	iNOS	(F)	GAGTCTTGGTGAAAGTGGTGTTC
		(R)	TTCCCTGTCTCAGTAGCAAAGAG
	IL-6	(F)	ACCACGGCCTTCCCTACTTC
		(R)	AGATTGTTTTCTGCAAGTGCATCA
	TNFα	(F)	CCAGACCCTCACACTCAGATC
		(R)	CACTTGGTGGTTTGCTACGAC
	IL-1β	(F)	GCTGCTTCCAAACCTTTGAC
		(R)	TTCTCCACAGCCACAATGAG
	IFNγ	(F)	GCTGACCTAGAGAAGACACATCAG
		(R)	GTGTGTAGCGTTCATTGTCTCAG
	IL-23a	(F)	GACTCAGCCAACTCCTCCAG
		(R)	GGCACTAAGGGCTCAGTCAG
	α_2 *integrin*	(F)	CCGGGTGCTACAAAAGTCAT
		(R)	GTCGGCCACATTGAAAAAGT
	α_4 *integrin*	(F)	CCCAGGCTACATCGTTTTGT
		(R)	CATGAATGGGGGTAAGGATG
	α_5 *integrin*	(F)	AGCGACTGGAATCCTCAAGA
		(R)	TGCTGAGTCCTGTCACCTTG
	α_6 *integrin*	(F)	AGCCCCAGGGACTTACAACT
		(R)	CTCTTGGAGCACCAGACACA
	α_M *integrin*	(F)	GACTCAGTGAGCCCCATCAT
		(R)	AGATCGTCTTGGCAGATGCT
	α_L *integrin*	(F)	CTGTTCTTTGGGGAGCAGAG
		(R)	CAGCCACATCAGTCAGCCTA
	α_V *integrin*	(F)	GGGTGATCATCTTGGCAGTT
		(R)	GAACTTGGAGCGGACAGAAG
	β_1 *integrin*	(F)	GCCAGGGCTGGTTATACAGA
		(R)	TCACAATGGCACACAGGTTT
	β_2 *integrin*	(F)	TGCCCTAGCTGGACTGTTCT
		(R)	TGATATCATCGGCTGGACAA
	β_3 *integrin*	(F)	TGTATCGTCTGTGCCTCTGC

		(R)	TTGCCATCCTCACCCTTAAC
	β_5 integrin	(F)	GGTTTCGGGTCTTTTGTTGA
		(R)	GCTTCCTCACTTCCTCGTTG
	CCR2	(F)	TCAGTTCATCCACGGCATAC
		(R)	TGACAAGGCTCACCATCATC
	CCR5	(F)	CGAAAACACATGGTCAAACG
		(R)	TTCCTACTCCCAAGCTGCAT
	CX3CR1	(F)	TTGGAACCATCTTCCTGTCC
		(R)	ACCAACAGATTTCCCACCAG
	Arginase1	(F)	AACTCTTGGGAAGACAGCAGAG
		(R)	GTAGTCAGTCCCTGGCTTATGG
	IL-10	(F)	AAATAAGAGCAAGGCAGTGGAG
		(R)	TCATTCATGGCCTTGTAGACAC
	Fizz	(F)	AGGATGCCAACTTTGAATAGGA
		(R)	AGTTAGCTGGATTGGCAAGAAG
	Mrc1	(F)	ATTCCGGTATCTGAACTGGCTA
		(R)	TGAGGGAATGATAAATGGGTTC
	MMP9	(F)	TCACACGACATCTTCCAGTACC
		(R)	CACCTCATTTTGGAAACTCACA
	β-actin	(F)	AGATTACTGCTCTGGCTCCTA
		(R)	CAAAGAAAGGGTGTAAAACG
	GAPDH	(F)	AAATGGTGAAGGTCGGTGTG
		(R)	AATCTCCACTTTGCCACTGC
Rat	*CCL2*	(F)	ATGCAGTTAATGCCCCACTC
		(R)	TTCCTTATTGGGGTCAGCAC
	IL-6	(F)	ATGTTGTTGACAGCCACTGC
		(R)	TAAGCCTCCGACTTGTGAAG
	TNFα	(F)	ACGATGCTCAGAAACACACG
		(R)	ATCCAGTGAGTTCCGAAAGC
	IL-1β	(F)	TGAAGCAGCTATGGCAACTG
		(R)	ATCTTTTGGGGTCTGTCAGC
	αSMA	(F)	AGGGAGTGATGGTTGGAATG
		(R)	GCCAGATCTTTTCCATGTCG
	PDGFRα	(F)	TGGAAGGCGCAGAAGCAATA
		(R)	TGCAAACGCGTGGTAAACAG
	Col 1a1	(F)	TGGTCCTCAAGGTTTCCAAG
		(R)	TTACCAGCTTCCCCATCATC
	Col 3a1	(F)	AGCTGGACCAAAAGGTGATG
		(R)	TCCAGTTAGCCCTGCAATTC
	MMP2	(F)	AGCTCCCGGAAAAGATTGAT
		(R)	TCCAGTTAAAGGCAGCGTCT
	MMP9	(F)	TTCAGAAGCAGCTGTCCCTG
		(R)	AGTCATCGATCACGTCTCGC
	MMP13	(F)	ACCCAGCCCTATCCCTTGAT
		(R)	ACATGAGGTCTCGGGATGGA

	Annexin A2	(F)	TCTGACTAACCGCAGCAATG
		(R)	TCAACAGGCCTAACATCACG
	AGPAT1	(F)	TGCTGCTCCACGTCAAATAC
		(R)	ACCTCCATCATTCCAAGCAG
	AGPAT2	(F)	TCAGCGGACAGAAGAAACTG
		(R)	TCCATGAGACCCATCATGTC
	β-actin	(F)	CCCATCTATGAGGGTTACGC
		(R)	TTTAATGTCACGCACGATTTC
Human	*ICAM-1*	(F)	GGCTGGAGCTGTTTGAGAAC
		(R)	ACTGTGGGGTTCAACCTCTG
	VCAM-1	(F)	TAAAATGCCTGGGAAGATGG
		(R)	GGTGCTGCAAGTCAATGAGA
	PECAM-1	(F)	ATGATGCCCAGTTTGAGGTC
		(R)	ACGTCTTCAGTGGGGTTGTC
	E-selectin	(F)	TGATGAGAGGTGCAGCAAGA
		(R)	GGATTCCAGGGCTGTACAGT
	P-selectin	(F)	CGAGGACTGCGTGGAGATAT
		(R)	ACAGGAGCAGGTGTAGTTCC
	β-actin	(F)	AGGCACTCTTCCAGCCTTCC
		(R)	GCACTGTGTTGGCGTACAGG

F: Forward, R: Reverse

Supplementary Fig. S1. Akt activation with conditioned media or AM treatment in stressed cardiomyocytes *in vitro*. (**a**) Western blot analysis of Akt activation in cardiomyocytes under hypoxia (1% O_2) with the specified conditioned medium as in Fig.1a (1 – 5). (**b**) Akt activation was observed in cardiomyocytes treated with conditioned medium from either primary hepatocytes (labeled primary) or the Huh7 cell line. (**c**) The effects of Huh7-conditioned medium at room temperature or boiled at 100°C for 10 min on Akt activation in cardiomyocytes after 12 hrs of treatment. (**d**) UV traces of anion exchange chromatography (left) followed by gel filtration (right) during protein purification. A blue line in the chromatograms denotes the UV traces. The UV value of fraction 3 in gel filtration is approximately 10 mAU. Stimulation either with fraction A4, A5, or A6 in anion exchange chromatography activates Akt in cultured cardiomyocytes. Huh7-CM, Huh7-derived conditioned medium. (**e**) Akt activation is observed in cardiomyocytes after treatment with 10 μg/ml of AM, but not bikunin. (**f**) Gender differences in AM mRNA expression in the liver (left) and AM protein distribution in the LV (right) of 6-week-old wild type mice.

Supplementary Fig. S2. AM protein distribution in mouse MI and AM serum concentration in acute MI patients. (**a**) Differential AM protein (green) and granulocyte marker LY-6G (red) localization in the BZ at day 3 in mouse MI. Scale bar, 100 μm. (**b**) AM serum concentration in control subjects and acute MI patients.

Supplementary Fig. S3. The effects of AM on mRNA expression and function in CFBs, MQs, and endothelial cells *in vitro*. (**a**) αSMA mRNA expression in CFBs after AM administration with TGFβ1 stimulation. *$p < 0.05$, compared to the negative control. (**b**) Changes in the mRNA expression of integrins (various alpha and beta subunits) and chemokine

receptors (CCR2, CCR5, CX3CR1) in J774 MQs. (**c**) Representative phase contrast images of AM-induced cell aggregation in J774 MQs after 24 hours. Scale bars, 100 μm. (**d**) Changes in the mRNA expression of ICAM-1, iNOS, and IL-6 in bone marrow-derived macrophages with AM stimulation. *p < 0.05, compared to 0 hrs. (**e**) Changes in mRNA expression of cell adhesion-related genes (ICAM-1, VCAM-1, PECAM-1, E-selectin, P-selectin) in human umbilical vein endothelial cells. *p < 0.05, compared to 0 hrs. (**f**) Tube formation assay in human umbilical vein endothelial cells after AM stimulation (n = 4 per group).

Supplementary Fig. S4. The effects of intramyocardial AM administration on cardiac function in mouse MI. (**a**) Retention period of native human AM protein injected into the BZ. The representative bright filed image under an optical microscope (MI day 1) and AM-immunostained images under an immunofluorescent microscope (day 2 and day 4) are shown. The native AM protein is brown-colored as described previously. Scale bars, 500 μm and 100 μm (higher magnification). (**b**) Representative photographs of cardiac rupture at day 3 in the MI + AM group. The dotted line and arrow head indicate the IZ edge and rupture site, respectively. (**c**) Echocardiographic parameters and heart weight to body weight ratio at 2 weeks post-treatment. *p < 0.05, compared to the sham group; **p < 0.05, compared to the MI + PBS group. Body weight, BW; heart weight, HW.

Supplementary Fig. S5. Screening of AM receptor. (**a**) Immunocoprecipitation of AM with annexin A2 and vimentin. (**b**) Confirmation of siRNA or overexpression of annexin A2 in CFBs *in vitro*. CFBs were collected at 48 hrs after transfection. *p < 0.05, compared to the control. (**c**) IL-1β mRNA expression in AM-stimulated CFBs after treatment with 5 μg/ml of a blocking antibody, siRNA, or overexpression of annexin A2 for 8 hrs. *p <0.05, compared to the negative

control. (**d**) Protein-lipid overlay assay using a sphingolipids strip. S1P, sphingosine-1-phosphate; SM, sphingomyelin; SPC, sphingosylphosphorylcholine; LPA, lysophosphatidic acid; GM1, monosialoganglioside; GD3, disiaganglioside; LPC, lysophosphocholine; PC, phosphatidylcholine. (**e**) Confirmation of AGPAT1 or AGPAT2 siRNA in CFBs. *$p < 0.05$, compared to the AM + control siRNA group. (**f** and **g**) Changes in IL-1β and MMP9 mRNA expression in AM-stimulated CFBs after treatment with AGPAT1 or AGPAT2 siRNA (**f**) and simultaneous AGPAT1 and AGPAT2 siRNAs (**g**). *$p < 0.05$, compared to the negative control; **$p < 0.05$, compared to the AM + control siRNA group. In (**g**), ctl, control; 1, AGPAT1; 2, AGPAT2; NS, not significant.

Supplementary Fig. S6. The effects of short-term, systemic delivery of a selective DGKα inhibitor in mouse MI. (**a** and **b**) Echocardiographic parameters (**a**), systolic blood pressure, heart rate, and HW to BW ratio (**b**) at 2 and 4 weeks in the sham or MI groups treated with vehicle, low or high dose of CU-3. *$p < 0.05$, compared to the sham group; **$p < 0.05$, compared to the MI + vehicle group. (**c**) Survival curve of each treatment group. (**d**) Representative immunofluorescent images showing MMP9 protein expression (green) in the I + BZs at day 3. Scale bars, 60 μm. (**e**) NFκB p65 activation (upper) and total MMP activity (lower) in the I + BZs at day 7 post-treatment analyzed by western blot and zymography, respectively.

Supplementary Fig. S1

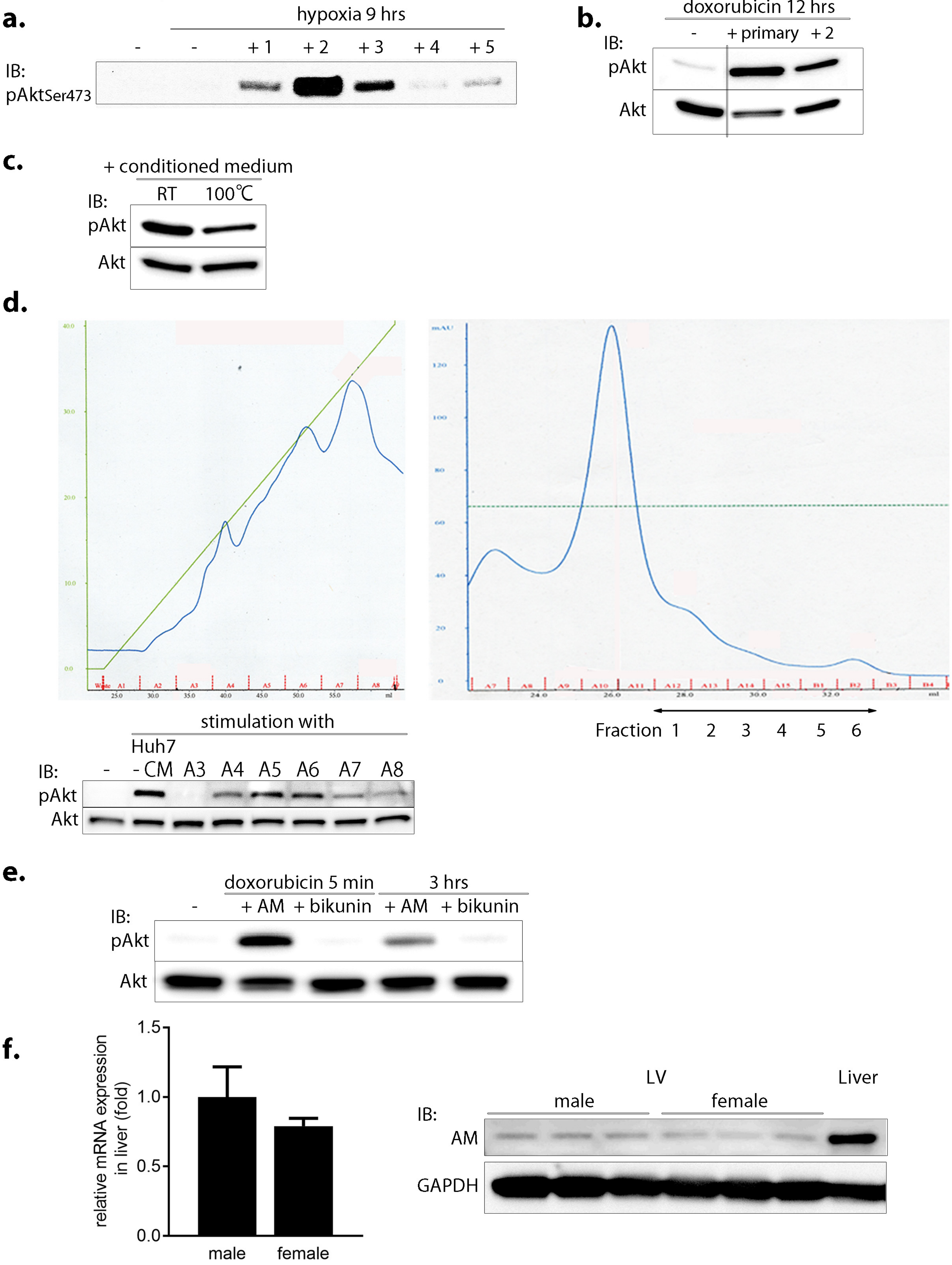

Supplementary Fig. S2

a.

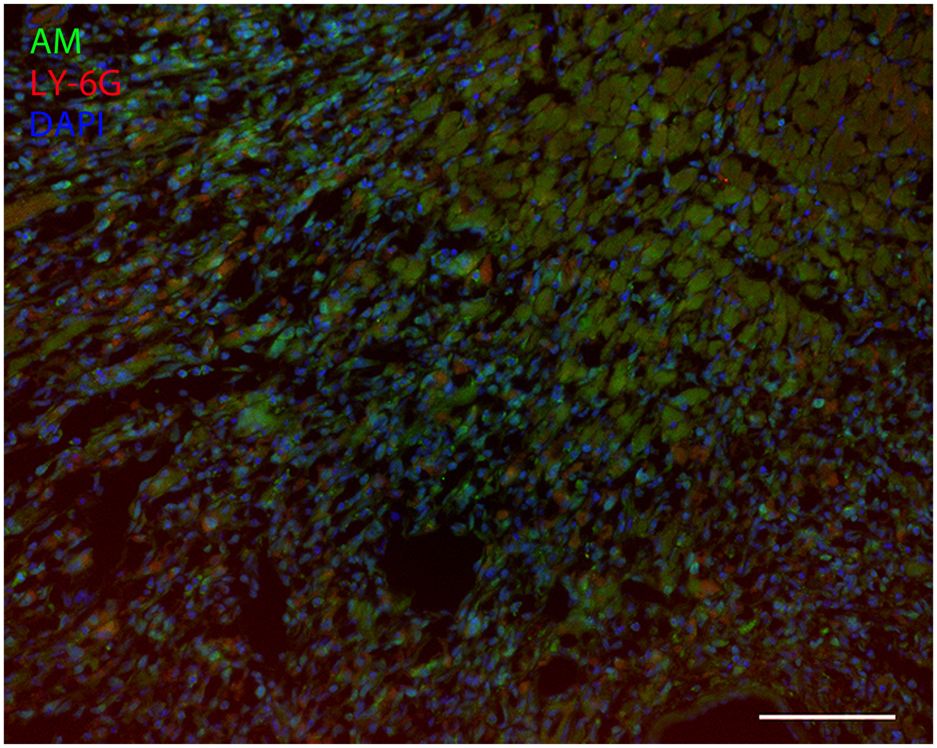

b.

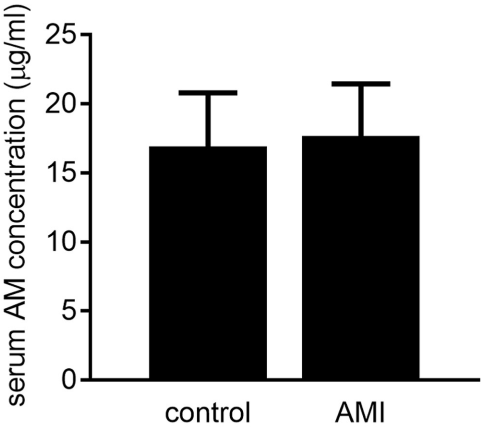

Supplementary Fig. S3

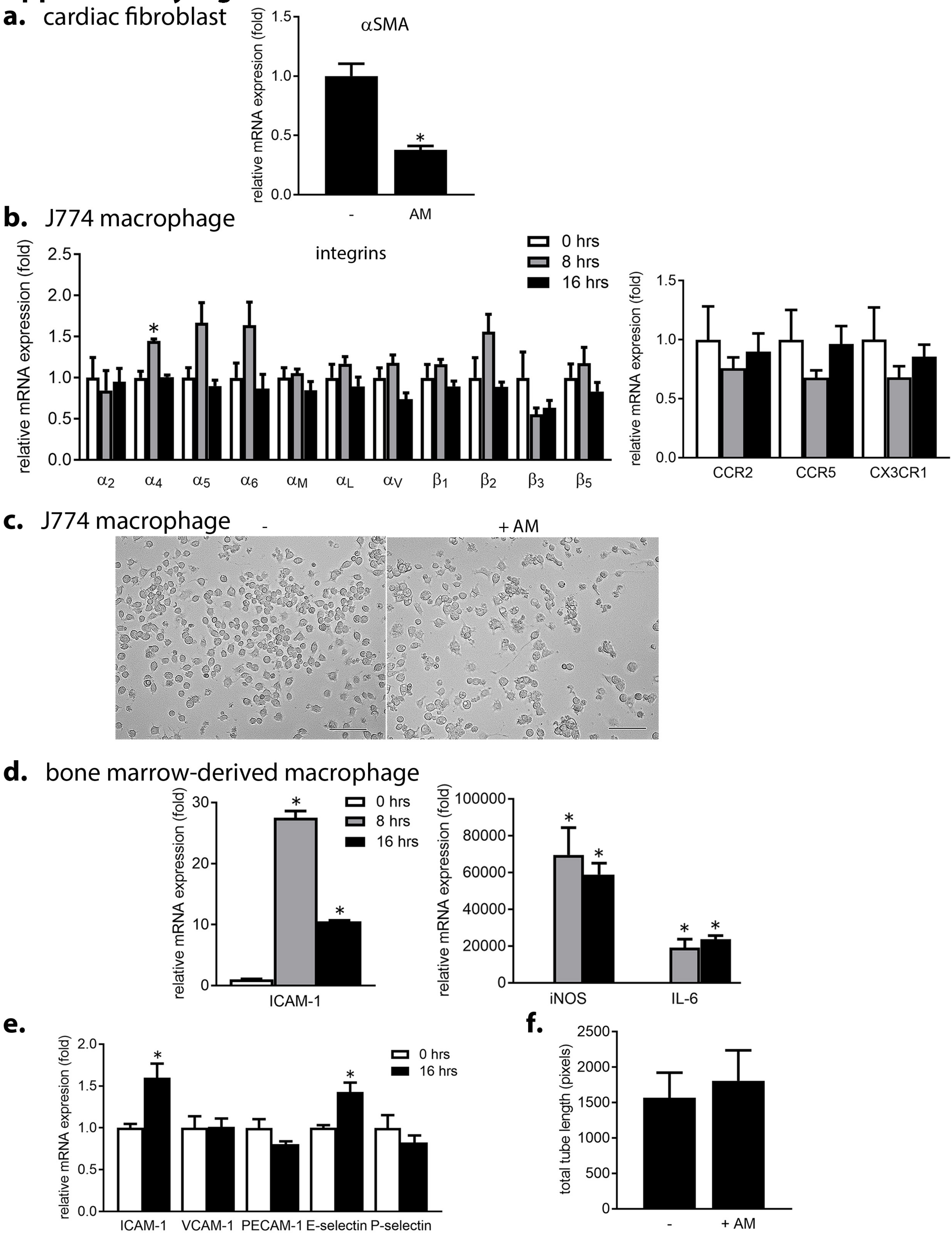

Supplementary Fig. S4

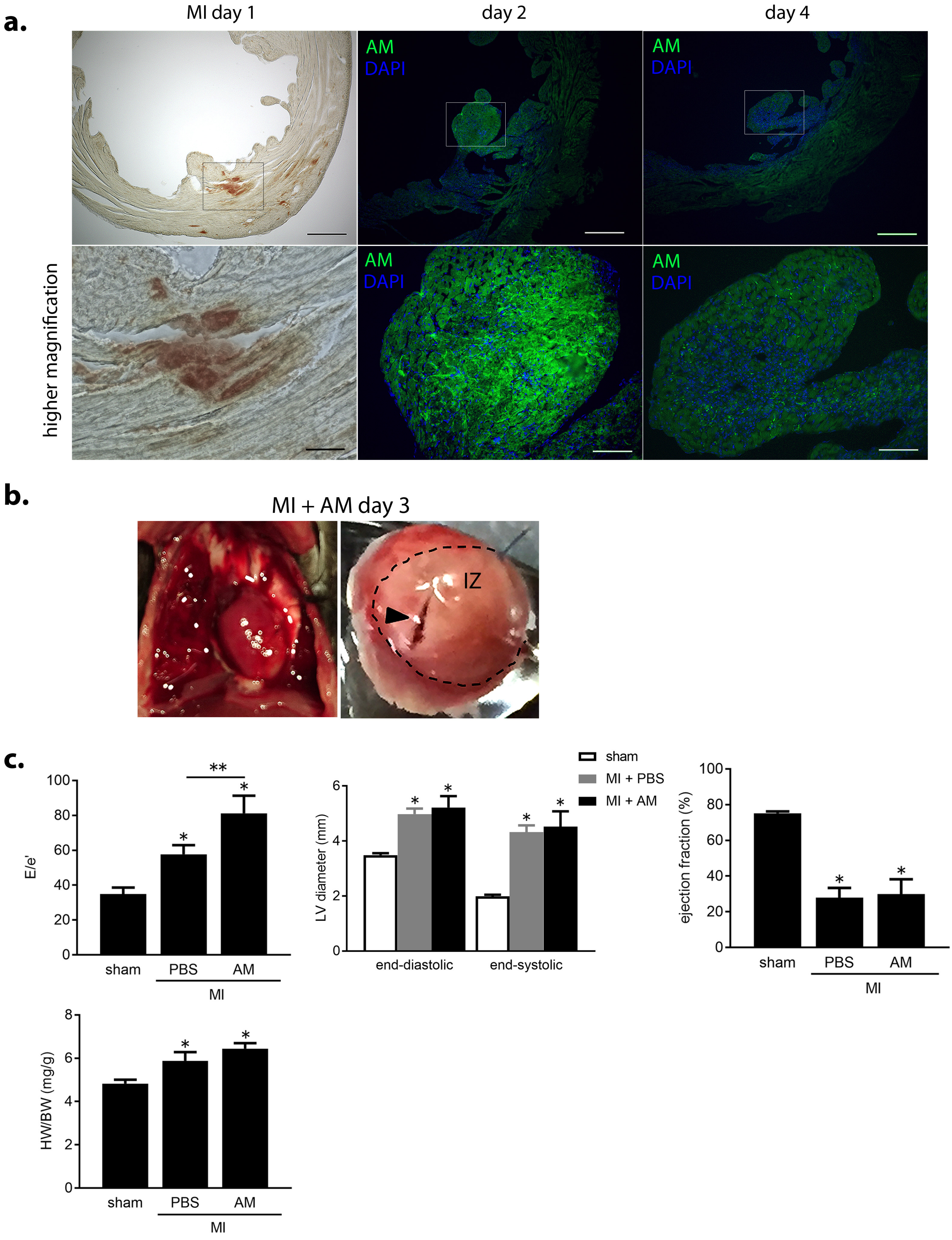

Supplementary Fig. S5

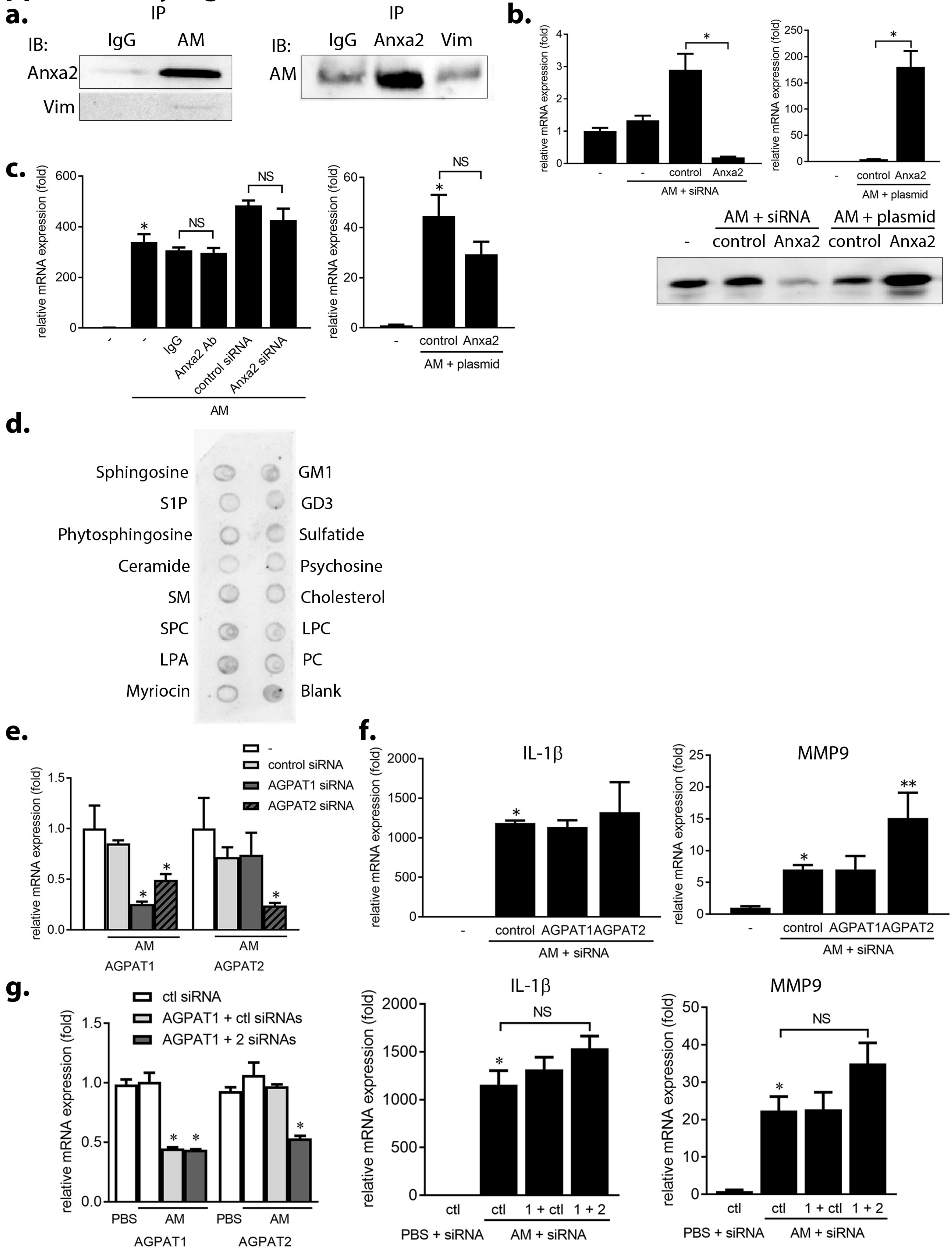

Supplementary Fig. S6

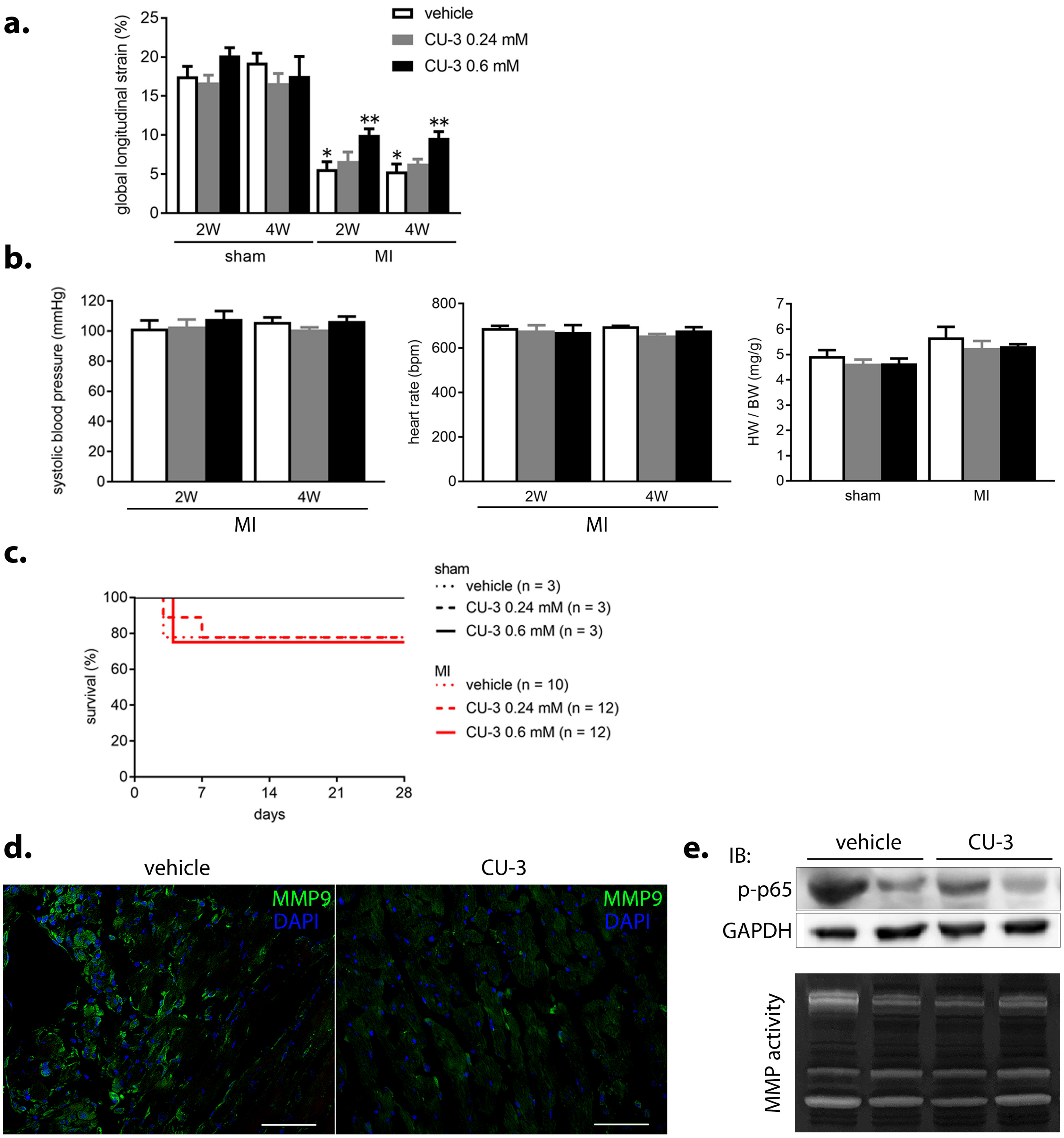

Supplement: Supplementary file 1 — Supplementary Information [file 41598_2018_35194_MOESM1_ESM.pdf]
